# Occurrence, diversity and community structure of culturable atrazine degraders in industrial and agricultural soils exposed to the herbicide in Shandong Province, P.R. China

**DOI:** 10.1186/s12866-016-0868-3

**Published:** 2016-11-08

**Authors:** Dmitry P. Bazhanov, Chengyun Li, Hongmei Li, Jishun Li, Xinjian Zhang, Xiangfeng Chen, Hetong Yang

**Affiliations:** 1 Key Laboratory for Applied Microbiology of Shandong Province, Ecology Institute (Biotechnology Center) of Shandong Academy of Sciences, Jinan, Shandong Province People’s Republic of China; 2 Biology Institute of Shandong Academy of Sciences, Jinan, Shandong Province People’s Republic of China; 3 Shandong Provincial Analysis and Test Center of Shandong Academy of Sciences, Jinan, Shandong Province People’s Republic of China

**Keywords:** Atrazine-degrading bacteria, Diversity, Soil bacterial communities, *Arthrobacter*, *Gulosibacter*, *Nocardioides*, *Pseudomonas*, *trzN*, *atz* genes

## Abstract

**Background:**

Soil populations of bacteria rapidly degrading atrazine are critical to the environmental fate of the herbicide. An enrichment bias from the routine isolation procedure prevents studying the diversity of atrazine degraders. In the present work, we analyzed the occurrence, diversity and community structure of soil atrazine-degrading bacteria based on their direct isolation.

**Methods:**

Atrazine-degrading bacteria were isolated by direct plating on a specially developed SM agar. The atrazine degradation genes *trzN* and *atzABC* were detected by multiplex PCR. The diversity of atrazine degraders was characterized by enterobacterial repetitive intergenic consensus-PCR (ERIC-PCR) genotyping followed by 16S rRNA gene phylogenetic analysis. The occurrence of atrazine-degrading bacteria was also assessed by conventional PCR targeting *trzN* and *atzABC* in soil DNA.

**Results:**

A total of 116 atrazine-degrading isolates were recovered from bulk and rhizosphere soils sampled near an atrazine factory and from geographically distant maize fields. Fifteen genotypes were distinguished among 56 industrial isolates, with 13 of them representing eight phylogenetic groups of the genus *Arthrobacter*. The remaining two were closely related to *Pseudomonas alcaliphila* and *Gulosibacter molinativorax* and constituted major components of the atrazine-degrading community in the most heavily contaminated industrial plantless soil. All isolates from the adjacent sites inhabited by cogon grass or common reed were various *Arthrobacter* spp. with a strong prevalence of *A. aurescens* group. Only three genotypes were distinguished among 60 agricultural strains. Genetically similar *Arthrobacter ureafaciens* bacteria which occurred as minor inhabitants of cogon grass roots in the industrial soil were ubiquitous and predominant atrazine degraders in the maize rhizosphere. The other two genotypes represented two distant *Nocardioides* spp. that were specific to their geographic origins.

**Conclusions:**

Direct plating on SM agar enabled rapid isolation of atrazine-degrading bacteria and analysis of their natural diversity in soil. The results obtained provided evidence that contaminated soils harbored communities of genetically distinct bacteria capable of individually degrading and utilizing atrazine. The community structures of culturable atrazine degraders were habitat-specific. Bacteria belonging to the genus *Arthrobacter* were the predominant degraders of atrazine in the plant rhizosphere.

**Electronic supplementary material:**

The online version of this article (doi:10.1186/s12866-016-0868-3) contains supplementary material, which is available to authorized users.

## Background

First registered in Switzerland and the United States in 1958, atrazine has soon become one of the world’s best-selling and heavily applied herbicides [1]. Nowadays, atrazine is a commonly detected contaminant of soils, underground and surface streams and basins [2–9]. Research performed during the first three decades of atrazine application indicated that the herbicide, like other chlorinated *s*-triazines, was poorly biodegradable through N-dealkylation of side chains by microbial hydrolases with low specific activities and subsequent dechlorination of the intermediates [10]. Atrazine was considered moderately persistent in soils; with a half-life estimate of about 2 months [11, 12]. However, atrazine residues and metabolites are detectable in soil for years [11, 13–15] and even decades [16] after the herbicide application.

Since the mid-nineties of the 20^th^ century, a rapid degradation of atrazine has been revealed in soils continuously exposed to the herbicide at various geographical locations [15]. The half-life of atrazine in such adapted soils can be as short as 1–3.5 days [17], causing reduced efficacy of weed control. From the ecological point of view, the enhanced degradation substantially decreases the harmful consequences of atrazine application by reducing both its conversion to stable dealkylated metabolites and the leaching of atrazine and its metabolites to deep soil horizons [15].

The enhanced atrazine degradation in soils was linked with the abundance of bacteria that had acquired novel metabolic abilities [17, 18] first discovered in *Pseudomonas* sp. strain ADP [19]. This bacterium mineralized atrazine through its dechlorination and further conversion of hydroxyatrazine to cyanuric acid by cleavage of the alkylamino side chains [20]. The genes *atzABC*, coding enzymes for the 3-step conversion were found to be highly conserved in phylogenetically distant bacteria [21]. While AtzB and AtzC are still the only known hydrolases for the specific transformation of hydroxyatrazine to cyanuric acid, an alternative, coded by the gene *trzN*, atrazine chlorohydrolase has been discovered in *Nocardioides* sp.190 [22] and later detected in some other bacteria [15]. Since cyanuric acid has been found to be readily degraded by a large number of microorganisms under a wide variety of natural conditions [10, 23, 24], it is arguable that the presence of bacteria harboring functional genes *atzABC* or *trzN* and *atzBC* is critical for the enhanced mineralization of atrazine in soils.

According to the review of Krutz et al. [15], atrazine-utilizing bacteria bearing the genes *atzA* or *trzN* occur worldwide (except Antarctica) and belong to four genera within the phylum *Actinobacteria* (*Arthrobacter*, *Clavibacter*, *Nocardia*, *Nocardioides*) and ten genera within the phylum *Proteobacteria* (*Agrobacterium, Alcaligenes, Herbaspirillum, Pseudaminobacter, Pseudomonas, Polaromonas, Ralstonia, Rhizobium, Sinorhizobium, Stenotrophomonas*). Among the listed bacteria, several *Arthrobacter* sp. strains [25–28] and *Pseudomonas* sp. AD39 [28] were isolated from sewage systems or heavily polluted soils at atrazine manufacturing plants in China. Similar atrazine degraders were later isolated from Chinese agricultural soils at several geographically distant locations, and all the taxonomically characterized agricultural isolates, except *Pseudomonas stutzeri* SA1 [29], belonged to the genus *Arthrobacter* [26, 30–34]. Such a relatively narrow taxonomic range of atrazine degraders isolated in China might be explained by the fact that their isolation targeted the selection of highly effective strains for treatment of wastewaters or contaminated soils rather than the analysis of atrazine-degrading microbial communities. The geographical occurrence, abundance and diversity of atrazine degrading bacteria remained poorly characterized. Among the known atrazine degrading bacteria, including the Chinese strains, a vast majority was isolated after procedures of repeated subculturing in media containing atrazine as a sole nitrogen or carbon source, principally similar to those first described by Mandelbaum et al. [35]. Selection of the isolates during the enrichment and probable elimination of many other atrazine-degrading members of microbial communities prevented studying the natural diversity and community structure of atrazine degraders in soils. Also, the isolation results may be affected by horizontal transfer of atrazine degradation genes during repeated enrichment. Owing to the genetic rearrangements, atrazine-mineralizing strains can be recovered even from the mixed cultures originated from soils which harbor no bacteria utilizing atrazine individually [19].

Shandong Province has been a region of intensive atrazine application for more than 20 years. Moreover, atrazine factories of several companies are located in Shandong Province, causing soil exposure to the herbicide of various rates, duration and periodicity, thus favoring the differential development of atrazine-degrading microbial communities in the surrounding area. The present work aimed to analyze the occurrence, phylogenetic diversity and community structure of atrazine-degrading bacteria in industrially contaminated and agricultural soils in Shandong Province based on their isolation by direct plating on a selective agar medium. Additionally, the occurrence of atrazine degraders was assessed by a direct detection of the genes for enhanced atrazine degradation in soil DNAs by conventional PCR.

## Results and discussion

### Soil properties

Soil samples were collected near an atrazine factory in Weifang Prefecture and from maize fields in four prefectures of Shandong Province during spring and summer of 2013 (Table 1, Additional file 1: Figure S1). Additionally, soil not previously treated with atrazine was collected from non-arable hillside area at the Eastern Campus of Shandong Academy of Sciences, Jinan. The detailed soil characteristics were analyzed in the Laboratory of Environmental Analysis of the Shandong Provincial Analysis and Test Center (Additional file 2: Table S1). Among three industrial sites, a high concentration of chloride (3200 ± 5 mg kg^−1^), increased contents of available nitrogen (437 ± 22 mg kg^−1^) and sodium (942 ± 94 mg kg^−1^), and the highest atrazine concentration (2091.3 ± 60.-0 μg kg^−1^, Table 1) were detected in D3 soil, sampled near a control well of the factory wastewater pipeline. In soil taken at D6 site, a distance of about 150 m from D3, the atrazine content was nearly 2000 times lower (Table 1). All agricultural soils had a history of atrazine use for at least 5 years and received atrazine treatment from 3 to 7 weeks before sampling dates (Table 1). Low or trace amounts of atrazine were found in all agricultural soils tested and, surprisingly, in the “wild” S1 soil.

**Table 1:** Characteristics of sampling sites

Sites	Geographic coordinates	Atrazine history^a^	Plants/Crop rotation	Sowing dates^a^	Atrazine treatment date^a^	Atrazine (active ingredient) rate^a, b^, g ha^−1^	Atrazine residues, μg kg^−1^	Sampling date	Plant growth stage
S1	N: 36.66361 E: 117.26151	No treatments for ≥ 20 years	Miscellaneous wild herbs	n/a	n/a	n/a	0.21 ± 0.03	April 08, 2013	dormancy
D3	N: 37.12418 E: 119.07188	7 years, industrial contamination	Plantless soil	n/a	n/a	n/a	2091.3 ± 60.0	May 29, 2013	n/a
D5	N: 37.12544 E: 119.07162	7 years, industrial contamination	Cogon grass (*Imperata cylindrica* (Linn.) Beauv.)	n/a	n/a	n/a	NA^c^	May 29, 2013	3–5 leaves
D6	N: 37.12539 E: 119.07087	7 years, industrial contamination	Common reed (*Phragmites australis* (Cav.) Trin. ex Steud.)	n/a	n/a	n/a	1.05 ± 0.13	May 29, 2013	2–3 leaves
TD(a)	N: 35.96921 E: 117.06004	≥20 years	Maize/ annual rotation with winter wheat	June 05–10	June 25–30	780	0.43 ± 0.06	July 22, 2013	9 leaves
TD(b)	N: 35.96980 E: 117.05999	≥20 years	Maize/ annual rotation with winter wheat	June 01–05	June 20–25	780	TA^c^	July 22, 2013	11 leaves
DnW	N: 35.07492 E: 115.64059	≥5 years	Maize/ annual rotation with winter wheat	June 01–05	June 20–25	450	0.65 ± 0.08	July 22, 2013	10 leaves
DnL	N: 35.09441 E: 115.66002	≥5 years	Maize/ annual rotation with winter wheat	June 01–05	June 20–35	450	0.08 ± 0.02	July 23, 2013	11 leaves
GD	N: 36.85381 E: 116.37587	≥10 years	Maize/ annual rotation with winter wheat	June 18	July 04–05	950	TA	August 14, 2013	pollen shed beginning
WS	N: 37.23022 E: 116.11851	≥15 years	Maize/ annual rotation with winter wheat	June 18	June 20	540	0.10 ± 0.02	August 14, 2013	pollen shed beginning

^a^The information was received from specialists of the local agriculture bureaus, managers of the farms, atrazine factory and academy campus

^b^In 2013

^c^ *NA* not analyzed, *TA* trace amount (0.01 < TA < 0.05 μg kg^−1^), *n/a* not applicable

### Detection, enumeration and isolation of atrazine-degrading bacteria by direct plating

The enrichment procedure [35] was believed to be essential for isolation of atrazine-degraders from soil [15]. The analyses of the literature allowed us to find four examples of a direct isolation of atrazine-utilizing bacteria [26, 36–38]. In three of the cases, soils used as isolation sources were previously exposed to extremely high (29 g kg^−1^ of soil [36]) or moderate (1.5 mg kg^−1^ [37, 38]) doses of atrazine, that could be deemed an alternative method of enrichment. Three-week incubation was required before colonies with clearing halos indicating degradation of atrazine were observed [26, 37, 38]. It was supposed that selective advantage for culturable atrazine-degrading bacteria in all isolation media used so far was reduced due to the hydrophobicity of atrazine. Therefore, Tween 80 was included in SM agar aiming to improve atrazine bioavailability. Addition of the surfactant provided perfect visual homogeneity to atrazine suspension in SM agar and enhanced colony growth of the reference atrazine-degrading strain *Arthrobacter* sp. SD41.

The occurrence of atrazine degraders was studied in industrial and agricultural soils that were differentially exposed to atrazine. The maize fields were normally treated with a low atrazine rate (Table 1) only once a year, therefore low densities of atrazine degraders were expected. Krutz et al. [18] demonstrated that the rhizosphere population of atrazine degraders was nearly twice that in a bulk soil. In order to increase the probability of detection the occurrence of atrazine-utilizing bacteria was analyzed in rhizosphere fractions of the agricultural soils sampled.

Direct plating on SM agar revealed atrazine-degrading bacteria in all samples of industrially contaminated and agricultural soils (Table 2). Colonies with typical clearing zones were visible by the end of the 3^rd^ incubation day (Additional file 3: Figure S2). Additional atrazine-degrading colonies appeared in most samples during further incubation, while some of the old colonies overlapped.

**Table 2:** Detection and enumeration of atrazine-degrading bacteria in soils

Sampling site	Soil^a^	Min-max population densities, CFU g^−1^ of dry soil		Percentage of atrazine degraders in the culturable population
		Culturable bacteria on TY agar	Atrazine-degrading bacteria on SM agar	
D3	I, B	5.8 × 10^8^–6.4 × 10^8^	2.9 × 10^7^–3.5 × 10^7^	4.5–6.0 %
D5	I, B	8.2 × 10^6^–8.6 × 10^6^	1.6 × 10^6^–2.4 × 10^6^	18.6–29.3 %
	I, R	8.8 × 10^7^–1.2 × 10^8^	1.1 × 10^7^–2.9 × 10^7^	9.1–33.0 %
D6	I, B	1.6 × 10^7^–2.0 × 10^7^	5.7 × 10^4^–7.4 × 10^4^	0.29–0.46 %
	I, R	6.1 × 10^8^–9.9 × 10^8^	3.6 × 10^5^–2.2 × 10^6^	0.05–0.23 %
TD(a)	A, R	6.5 × 10^8^–9.9 × 10^8^	1.3 × 10^3^–1.3 × 10^4^	0.0002–0.0014 %
TD(b)	A, R	NE^b^	1.8 × 10^3^–5.2 × 10^3^	NE
DnW	A, R	NE	1.3 × 10^3^–2.8 × 10^3^	NE
DnL	A, R	NE	8.0 × 10^2^–4.5 × 10^3^	NE
GD	A, R	4.8 × 10^8^–7.9 × 10^8^	1.4 × 10^4^–7.5 × 10^4^	0.0035–0.0095 %
WS	A, R	NE	3.0 × 10^2^–2.0 × 10^3^	NE

^a^ *I* industrial, *A* agricultural, *B* bulk, *R* rhizosphere. ^b^ *NE* not evaluated

The highest population densities of atrazine degrading bacteria were detected in D3 and D5 soils (Table 2). In the latter, both in bulk soil and in the rhizosphere of cogon grass, atrazine degraders accounted for nearly 1/3 of the recoverable bacterial population. The densities of atrazine degrading bacteria in the rhizosphere of cogon grass (sample D5) and common reed (sample D6) were about ten times higher than those in the bulk soils.

Populations of atrazine-degrading bacteria in the maize rhizosphere varied from 10^2^ to 10^4^ CFU g^−1^ soil (Table 2), indicating that they constituted a minor component of the microbial communities. In general, the population densities of atrazine degraders calculated from direct plate counts were in agreement with the data obtained by a radiological most probable number method for rhizosphere of maize growing in atrazine-adapted soils [18].

**Fig. 1:**
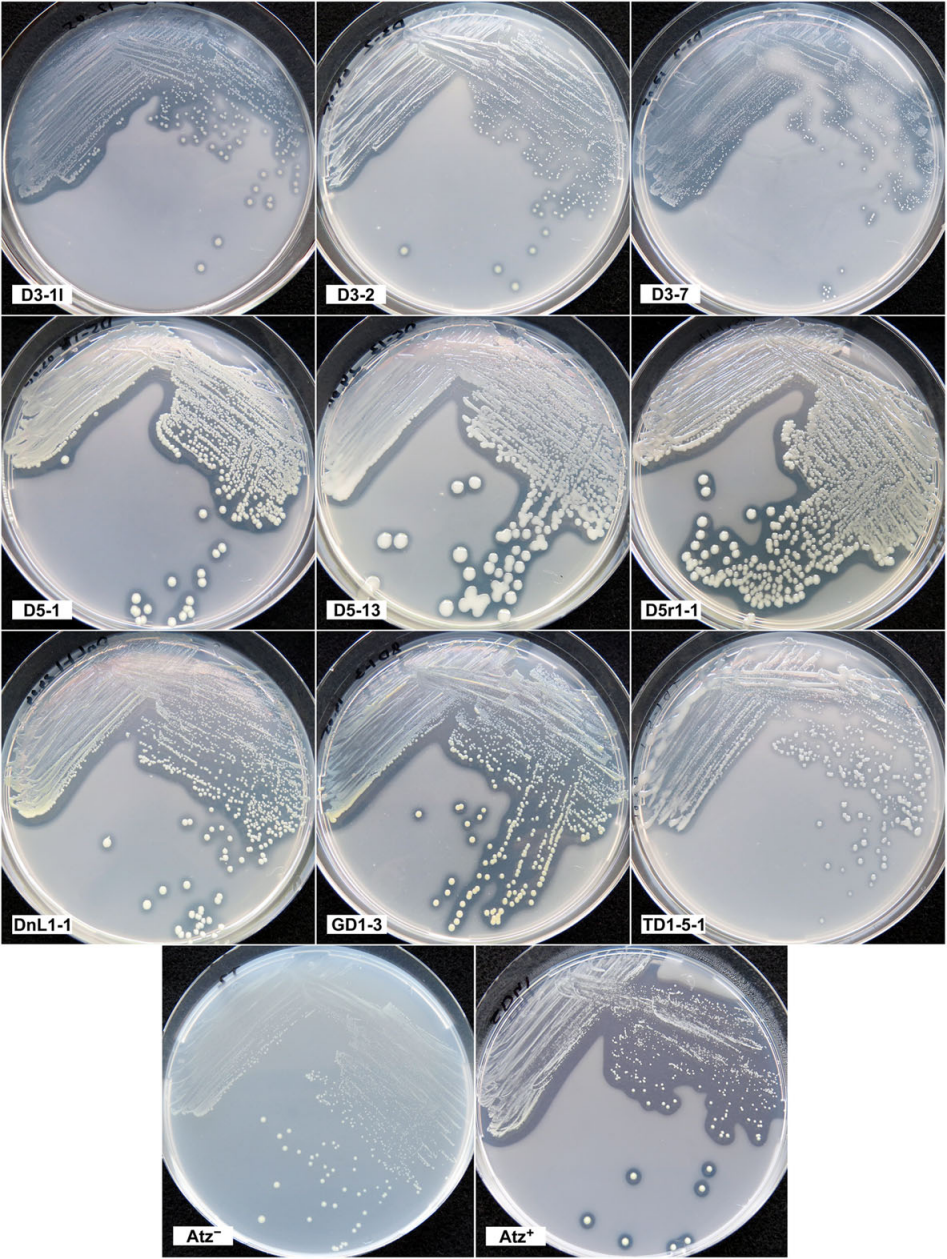
Cultures of some atrazine degrading isolates on SMY agar. Age of the cultures was 5 days (D3-1l, D3-2, D5-1, D5-13, D5r1-1, DnL1-1) or 7 days (D3-7, GD1-3, TD1-5-1). (Atz^−^) *A. ureafaciens* CGMCC 1.1897^T^, a negative control strain; (Atz^+^) the reference atrazine-degrading strain *Arthrobacter* sp. SD41

In sum, 116 strains of atrazine degraders were isolated by direct plating of soil dilutions. Among these, 56 strains were derived from samples of industrially contaminated soil, and 60 strains originated from agricultural soils. Six to 26 strains were isolated from each of the sampling sites. The isolates varied in streak and colony morphologies and colony growth rate (Fig. 1), indicating that direct plating on SM agar allowed the isolation of diverse bacteria showing different culturability.

### ERIC-PCR genotyping of atrazine-degrading strains

All the 116 atrazine degrading isolates were genotyped by Rep-PCR using primer ERIC2. As a result, 17 ERIC types designated A-Q (Table 3, Additional file 4: Figure S3) were distinguished. Analysis of 56 cultures isolated from the industrially contaminated soils (D samples) allowed discrimination between 15 different ERIC types. Among these, ERIC type A was strongly predominant at sites D5 and D6, both in the rhizosphere and bulk soil (Table 3). Besides type A, two other ERIC types of atrazine-utilizing bacteria were found at both sites. The second largest, ERIC type L, comprised bacteria isolated from bulk soil. In contrast, bacteria of ERIC type E were isolated from the rhizosphere (1 isolate from each of the sites). In addition to the ERIC types common for these two sites, seven minor types (B, I, J, K, M, N and Q) of atrazine degraders were found only at site D5. Three ERIC types (F, G and H) were detected among nine isolates from D3 soil. No ERIC types found among isolates from D5 and/or D6 samples were detected among the bacteria isolated from D3 soil*.*

**Table 3:** ERIC types of atrazine-degrading isolates and their geographic occurrence

ERIC types	Number of atrazine degrading strains isolated from soils													
	D3	D5			D6			TD(a)	TD(b)	DnW	DnL	GD	WS	Total
	I, B^a^	I, B	I, R	Total	I, B	I, R	Total	A, R	A, R	A, R	A, R	A, R	A, R	
A	0	5	9	14	3	12	15	0	0	0	0	0	0	29
B	0	0	2	2	0	0	0	10	6	6	8	9	8	49
C	0	0	0	0	0	0	0	3	4	0	0	0	0	7
D	0	0	0	0	0	0	0	0	0	0	0	4	2	6
E	0	0	1	1	0	1	1	0	0	0	0	0	0	2
F	2	0	0	0	0	0	0	0	0	0	0	0	0	2
G	5	0	0	0	0	0	0	0	0	0	0	0	0	5
H	2	0	0	0	0	0	0	0	0	0	0	0	0	2
I	0	1	0	1	0	0	0	0	0	0	0	0	0	1
J	0	1	0	1	0	0	0	0	0	0	0	0	0	1
K	0	1	0	1	0	0	0	0	0	0	0	0	0	1
L	0	3	0	3	3	0	3	0	0	0	0	0	0	6
M	0	1	0	1	0	0	0	0	0	0	0	0	0	1
N	0	1	0	1	0	0	0	0	0	0	0	0	0	1
O	0	0	0	0	0	1	1	0	0	0	0	0	0	1
P	0	0	0	0	0	1	1	0	0	0	0	0	0	1
Q	0	1	0	1	0	0	0	0	0	0	0	0	0	1
Total strains	9	14	12	26	6	15	21	13	10	6	8	13	10	116
Total ERIC types	3	8	3	10	2	4	5	2	2	1	1	2	2	17

^a^ *I* industrial, *A* agricultural, *B* bulk, *R* rhizosphere

Only three ERIC types were discriminated among 60 strains isolated from the maize rhizosphere soils, collected from six geographically distant fields. Forty seven strains fitted into ERIC type B, which was originally detected among isolates associated with cogon grass roots in D5 soil. ERIC type B atrazine-degrading bacteria were found in all replicate samples of maize rhizosphere soils from all field sites. The reference bacterium *Arthrobacter* sp. SD41 also belonged to ERIC type B.

The other two ERIC types of atrazine-utilizing bacteria found in the maize rhizosphere were represented by seven type C and six type D isolates. Strains of ERIC type C were isolated form all replicate samples taken at TD(a) and TD(b) sites. Strains of ERIC type D were found in all GD replicate samples and in two replicate samples from WS site. Bacteria of ERIC types C and D were not found among atrazine degraders originating from other sampling sites, suggesting a localized distribution.

Thus, ERIC-PCR genotyping of atrazine-degrading bacteria directly isolated from industrially contaminated soils revealed communities of genetically distinct strains individually utilizing atrazine as a sole nitrogen source. ERIC type A bacteria were identified as dominant in atrazine-degrading communities at sites D5 and D6 both in bulk and rhizosphere soils, while most isolates from site D3 were ERIC type G bacteria. Along with the absence of any common ERIC type, this indicated a general dissimilarity between the structures of atrazine degrading communities in soil D3 and soils D5-D6, despite the sampling sites being separated by only 150 m. Population densities of atrazine degraders in all three soils were high (Table 2), indicating high rates of atrazine inflow. Among the populations in bulk soils the highest density was detected at site D3, where atrazine content was nearly 2000 times higher than in D6 soil (Table 1). Assuming similar leaching rates at sites D3 and D6, the difference between the measured atrazine concentrations would result mainly from the balance between atrazine inflow and degradation. Therefore, the high concentration of atrazine in D3 soil indicated that the contamination rate exceeded the degradation potential of the bacterial community.

Concentration of available nitrogen in D3 soil was nearly ten times higher than in D5 and D6 soils, and similar to or even higher than that in common mineral microbiological media (437 ± 22 mg N kg^−1^ soil, Additional file 2: Table S1, is equivalent to 1680 mg NH_4_Cl kg^−1^ soil, or 6.0 g NH_4_Cl L^−1^ soil solution on the basis of 35 % water holding capacity and 80 % humidity of soil). It was obvious that D3 soil contained excess nitrogen and its availability did not limit bacterial growth. Therefore, atrazine seemed to stimulate the population growth of its degraders in D3 soil as a source of carbon and energy rather than of nitrogen. The excess nitrogen from the degraded atrazine could contribute to the pool of available nitrogen in soil. Thus, the specific genetic structure of the atrazine degrading community in D3 soil seemed to be caused mainly by its heavy contamination with atrazine and other components of the factory wastewater.

ERIC typing of atrazine degrading isolates from the maize rhizosphere revealed a narrow range of genotypes. And despite the possible presence of some minor genetic groups of atrazine degraders that were below the limit of detection, the contrast with the diversity observed in the industrially contaminated soils was striking. Bacteria of ERIC type B were the dominant or even the sole group of atrazine degraders detected in the maize rhizosphere at all agricultural sites investigated. This clearly indicated their prevalence on the large area of agricultural soils with a history of atrazine application. Taking into consideration that agricultural soils were treated with low doses of atrazine and only once a year, traits other than the ability to utilize atrazine seemed to contribute to the competence of ERIC type B bacteria in the maize rhizosphere.

### Detection of genes for atrazine degradation and atrazine-degrading capacity in the isolates

The multiplex PCR mixture contained four primer pairs allowing selective amplification of the genes *atzA*, −*B*, −*C* and *trzN* to generate fragments with predicted sizes of 432, 275, 626, and 196 base pairs respectively. One of the reference strains was *Arthrobacter* sp. SD41, bearing the genes *atzB*, −*C* and *trzN* [34]. Later the genes *atzA*, −*B* and –*C* were detected in strain *Pseudomonas* sp. D3-1l isolated in this work. Multiplex PCRs with cell lysates of strains SD41 or D3-1l as templates produced respective fragments of the predicted sizes at the wide range of T_a_ (Additional file 5: Figure S4), indicating robust detection of the genes *atzB*, −*C* and *trzN* in *Arthrobacter* sp. SD41 and the genes *atzA*, −*B* and –*C* in strain D3-1l. No production of unexpected fragments was detected.

The fragments produced in the multiplex PCRs were sequenced to verify their identity to the genes targeted. The resulting nucleotide sequences of *atzB* and -*C* from *Arthrobacter* sp. SD41 and *atzA* from D3-1l were deposited in GenBank (http://www.ncbi.nlm.nih.gov/genbank) under accession Nos. KP994320 - KP994322. The nucleotide sequence of a short *trzN* fragment from *Arthrobacter* sp. SD41 is provided in Additional file 6. Additionally, an amplified with the common primers C190-10/C190-11 fragment of *trzN* gene from *Arthrobacter* sp. SD41 was sequenced (Accession No. KP994319). BLAST search results demonstrated that the determined nucleotide sequences shared high identity (98–100 %) with respective atrazine degradation genes from other bacteria, including the genes *atzA*, −*B* and -*C* from *Pseudomonas* sp. ADP and *trzN*, *atzB* and –*C* from *Arthrobacter aurescens* TC1.

Besides strain D3-1l, the gene *atzA* was detected in all ERIC type G bacteria (Table 4, Additional file 7: Figure S5). All isolates belonging to other ERIC types contained *trzN* gene. The genes *atzB* and *atzC* were detected in all isolates except 12 strains of ERIC type B and three isolates of ERIC type L. Also, *atzB* gene was not found in strains D6r1-2 (ERIC type A) and D3-2 (ERIC type G).

**Table 4:** Detection of atrazine degradation genes in the isolates and ribosomal diversity of the strains representing separate ERIC types

ERIC type	Genes for atrazine degradation	Strains (GenBank Accession No.)^a^	Nearest type strain (GenBank Accession No.) [Percent similarity]^b^
A	*trzN atzBC*	**D5-2 (KF889364)**, D5-4, D5-9, D5-10, D5-14, **D5r1-1 (KF889366)**, D5r1-3, D5r1-4, D5r2-1, D5r2-3, D5rh3-1, D5r3-3, D5r3-4, D5r3-5, **D6-2 (KF889365),** D6-4, D6-5, **D6r1-1 (KF889367)**, D6r1-3, D6r1-4, D6r2-1, D6r2-2, D6r2-3, **D6r2-5 (KF889368)**, D6r3-1, D6r3-2, D6r3-3, D6r3-4	*Arthrobacter nitroguajacolicus* G2-1^T^ (NR_027199) [99.3–99.4 %]
	*trzN atzC*	D6r1-2	
B	*trzN atzBC*	**D5r1-2 (KF889369)**, TD1-1, TD1-2, TD1-3, TD1-4, TD2-2, **TD2-4 (KF889370)**, TD3-1, TD3-2, TD3-3, **TD4-1 (KF889371)**, TD 4–2, TD5-1, TD6-1, TD6-3; **DnW1-1 (KF889372)**, DnW1-2, DnW2-1, DnW2-2, DnW3-1, DnW3-2; **DnL1-1 (KF889373)**, DnL1-3, DnL2-1, DnL3-1, DnL3-3; **GD1-1 (KF889374)**, GD1-4, GD2-2, GD2-3, GD3-1, GD3-2, GD3-3, GD3-4, GD3-5; WS1-1, WS2-3	*Arthrobacter ureafaciens* NC^T^ (NR_029281) [99.5– 99.6 %]
	*trzN*	D5r2-2, TD2-1**,** TD6-2, DnL1-2, DnL2-2, DnL3-2, WS1-2, WS2-1, **WS2-2 (KF889375)**, WS2-5, WS3-1, WS3-2.	
C	*trzN atzBC*	TD1-5-1**, TD1-5-2 (KF889379)**, **TD2-3 (KF889376)**, **D4-3 (KF889377)**, **TD4-4 (KF889378)**, TD5-2, TD5-3	*Nocardioides panacihumi* Gsoil 616^T^ (NR_041518) [98.1–98.3 %]
D	*trzN atzBC*	GD1-2, **GD1-3 (KF889380)**, GD2-1, GD2-4, **WS1-3 (KF889381)**, WS2-4	*Nocardioides ganghwensis* JC2055 ^T^ (NR_025776) [99.8 %]
E	*trzN atzBC*	**D6rh3-5 (KF889382)**	*Arthrobacter sulfonivorans* ALL^T^ (NR_025084) [99.4–99.6 %]
	*trzN atzC*	**D5rh3-2 (KF889383)**	
F	*trzN atzBC*	D3-7, **D3-8 (KF889384)**	*Gulosibacter molinativorax* ON4^T^ (NR_025451) [100 %]
G	*atzABC*	**D3-1l (KF889385)**, D3-1s, D3-4, D3-5	*Pseudomonas alcaliphila* AL15-21^T^ (NR_024734) [99.1–99.3 %]
	*atzAC*	**D3-2 (KF889386)**	
H	*trzN atzBC*	**D3-3 (KF889387), D3-6 (KF889388)**	*Arthrobacter crystallopoietes* DSM 20117^T^ (NR_026189) [99.8 %]
I	*trzN atzBC*	**D5-3 (KF889389)**	*Arthrobacter crystallopoietes* DSM 20117^T^ (NR_026189) [99.6 %]
J	*trzN atzBC*	**D5-5 (KF889390)**	*Arthrobacter crystallopoietes* DSM 20117^T^ (NR_026189) [99.8 %]
K	*trzN atzBC*	**D5-7 (KF889391)**	*Arthrobacter crystallopoietes* DSM 20117^T^ (NR_026189) [99.7 %]
L	*trzN atzBC*	D5-12, **D5-13 (KF889392)**	*Arthrobacter oxydans* DSM 20119^T^ (NR_026236) [99.7–99.8 %]
	*trzN atzBC*	**D5-8 (KF889393)**	
	*trzN*	**D6-1 (KF889394),** D6-3, D6-3 s	
M	*trzN atzBC*	**D5-6 (KF889395)**	*Arthrobacter phenanthrenivorans* Sphe3^T^ (NR_074770) [99.9 %]
N	*trzN atzBC*	**D5-11 (KF889396)**	*Arthrobacter subterraneus* CH7^T^ (NR_043546) [99.8 %]
O	*trzN atzBC*	**D6r1-5 (KF889397)**	*Arthrobacter nitroguajacolicus* G2-1^T^ (NR_027199) [100 %]
P	*trzN atzBC*	**D6r2-4 (KF889398)**	*Arthrobacter nitroguajacolicus* G2-1^T^ (NR_027199) [99.8 %]
Q	*trzN atzBC*	**D5-1 (KJ010189)**	*Arthrobacter nitroguajacolicus* G2-1^T^ (NR_027199) [99.6 %]

^a^Names of the strains selected for sequencing of 16S rRNA genes and GenBank accession numbers are typed in bold. Letters in the name of each strain and numbers after letter D represent sampling sites listed in the Table 1. Letter “r” following D5 and D6 means that the strain has been isolated from the rhizosphere. The first digit in the names of strains originated from TD, DnW, DnL, GD and WS sites indicates replicate samples. Site TD(a) was represented by replicate samples 1–3, and site TD(b) – by replicate samples 4–6

^b^ According to BLAST, based on over 1235 bp nucleotide sequences of 16S rRNA genes

Atrazine-degrading capacity of isolates representing distinguished ERIC types was assessed in liquid medium SM25 with 25 mg L^−1^ atrazine as a sole nitrogen source. After 1 week incubation, HPLC-MS/MS analysis detected trace amounts of atrazine (from <0.003 to 0.09 μg L^−1^) in cultural liquids of the strains D5r1-1 (ERIC type A), DnL1-1 (ERIC type B), TD1-5-1 (ERIC type C), GD1-3 (ERIC type D), D3-1l (ERIC type G), D3-3 (ERIC type H), D5-13 (ERIC type L), D6r2-4 (ERIC type P) and D5-1 (ERIC type Q), indicating over 99.99 % degradation. The concentration of atrazine in the cultural liquid of strain D3-7 (ERIC type F) was reduced to 3567 ± 312 μg L^−1^, demonstrating nearly 85.7 % degradation. No significant degradation of atrazine was observed in the non-inoculated control.

Reported in numerous papers over the last 20 years, the dissolution of atrazine in liquid media or production of colonies with clearing halos on solid media with atrazine were always due to its rapid degradation by bacteria harboring *atzA* or *trzN* in combination with *atzB* and/or *atzC* [15]. The non-degradative microbial dissolution of atrazine and other chlorinated *s*-triazines has never been observed and seems to be impossible in principle due to the low solubility of these compounds in all known solvents. The results obtained in our study once again confirm that the production of clearing halos by growing colonies and the presence of the genes *trzN* and/or *atzABC* in the genome are robust indicators of the atrazine-degrading capacity in bacteria. Direct isolation of bacteria producing colonies with clearing halos on SM medium and detection of *trzN* and *atzABC* genes by multiplex PCR may be promising methods to facilitate ecological and biogeographical studies of atrazine degraders.

Our data demonstrated the presence of atrazine degraders harboring the gene *trzN* in all studied soils and clearly indicated their prevalence over *atzA*-harboring bacteria at all agricultural sites and at two industrially contaminated sites. Arbeli and Fuentes [39] reached a similar conclusion for Colombian agricultural soils based on the analysis of soil DNA isolated after previous “microcosm enrichment” of atrazine degraders. They hypothesized that atrazine degraders harboring *trzN* had ecological superiority over *atzA*-harboring bacteria, owing to the faster reaction rate of TrzN, its higher affinity for substrates and wider range of substrates degraded [39]. Interestingly, Arbeli and Fuentes did detect *atzA* gene in enrichment cultures derived from some of the soils and found that enrichment with atrazine as a sole carbon and nitrogen source was markedly more helpful for the detection of *atzA* than with atrazine as the only source of nitrogen. Because atrazine is a poor source of carbon and rich in nitrogen, the growth of degrading bacteria in media with atrazine as sole carbon and nitrogen source is limited by carbon [10]. In contrast to all other sites, bacteria harboring *atzA* gene were found to be a major or even dominating group in the atrazine degrading community of D3 soil, indicating that *trzN* had no ecological superiority over *atzA*. Unlike soils from all other sites investigated, D3 soil had the highest content of atrazine and an excess of available nitrogen, low content of organic carbon and contained no plant roots that could provide substances rich in carbon and energy. These conditions are similar to those in the cultures with atrazine as a sole carbon and nitrogen source, which favor the enrichment of *atzA*-bearing atrazine degraders. Thus, it is possible to hypothesize that the high contamination rate has influenced the structure of atrazine-degrading community in D3 soil by altering the selective advantage for the degraders harboring *trzN* or *atzA*.

### Sequencing of 16S rRNA genes and phylogenetic analysis

The 16S rRNA genes of 36 strains representing all the 17 distinguished ERIC types were sequenced (Table 4). Selected strains of the same ERIC type represented all the sites where bacteria belonging to this ERIC type were isolated. BLAST search results gave evidence that 26 strains representing 12 ERIC types, including predominant types A and B, belonged to the genus *Arthrobacter*. Bacteria of two other major ERIC types C and D were found to belong to the genus *Nocardioides.* Besides *Arthrobacter* and *Nocardioides* no other genera were detected among strains isolated from the maize rhizosphere. Isolates from the industrial soils (D samples) were somewhat more diverse taxonomically. Strains of ERIC types F and G were affiliated to the genera *Gulosibacter* (phylum *Actinobacteria*, class *Actinobacteria*, subclass *Actinobacteridae*, order *Actinomycetales*, suborder *Micrococcineae*, family *Microbacteriaceae*) and *Pseudomonas* respectively.

**Fig 2:**
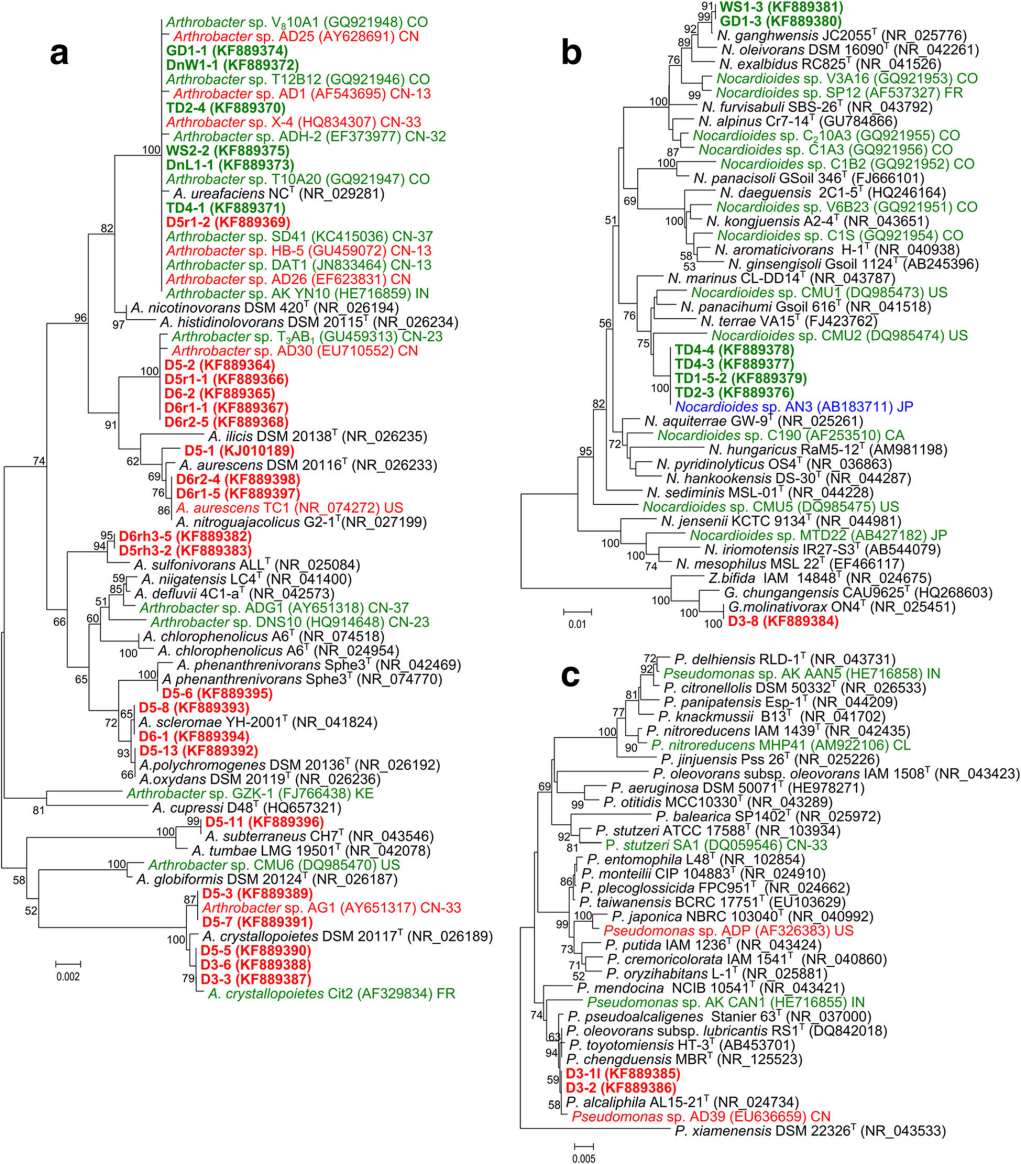
Neighbor-joining phylogenetic trees for atrazine-degrading bacteria belonging to the genera **a** *Arthrobacter*, **b** *Nocardioides* and *Gulosibacter*, **c** *Pseudomonas*, based on 16S rRNA gene sequences. Colors indicate atrazine-degrading bacteria isolated from industrial soils or sites of spill (*red*), agricultural soils (*green*), and riverbed sediment (*blue*). Names of the strains isolated in this work are printed in bold lettering. GenBank accession numbers are shown in parentheses. For known atrazine-degrading strains, countries of isolation, and for those isolated in China – provinces, are indicated by ISO 3166 codes. Bootstrap values (expressed as percentages of 1000 replications) greater than 50 % are shown at the branching points. There were a total of 1200 (**a**), 1206 (**b**) and 1258 (**c**) positions in the final datasets. Scale bars show substitutions per nucleotide position. Evolutionary analysis was conducted in MEGA5 [63]

To accurately define taxonomic positions of the isolates at the sub-genus level, a phylogenetic analysis was carried out based on the determined and reference nucleotide sequences of 16S rRNA genes. As a result, 26 representatives of 12 ERIC types belonging to the genus *Arthrobacter* were distributed into eight phylogenetic groups (Fig. 2a). It was found that all representative strains of ERIC type A constituted a tight cluster with known atrazine-degrading strains *Arthrobacter* sp. AD30 and *Arthrobacter* sp. T_3_AB_1_, which were isolated in China. *Arthrobacter* sp. T_3_AB_1_ was the only agricultural strain in the cluster and originated from maize field soil sampled in Nehe County, Heilongjiang Province [31], located about 1300 km north from sampling site D. The cluster contained no species type strains and was closely related to the phylogenetic group formed by strains D5-1 (ERIC type Q), D6r1-5 (ERIC type O), D6r2-4 (ERIC type P), the type strains of *A. aurescens*, *Arthrobacter nitroguajacolicus*, *Arthrobacter ilicis*, and the known atrazine-degrading bacterium *A. aurescens* TC1, isolated in the United States from soil heavily contaminated with atrazine due to an accidental spill [36]. The divergence between these two clusters exceeded the divergence between the species *A. aurescens*, *A. nitroguajacolicus* and *A. ilicis*, suggesting that bacteria of ERIC type A represented a separate genomospecies.

All seven strains selected from the largest group of ERIC type B isolates tightly clustered with the type strain of *Arthrobacter ureafaciens*, suggesting their affiliation with this species. This robust cluster also contained eight of the 12 Chinese atrazine-degrading strains of the genus *Arthrobacter* for which 16 rRNA gene nucleotide sequences of the proper length and quality have been determined and published [25, 27, 30, 33, 34, 40, 41], 1 isolate originating from India [42] and 3 Colombian atrazine degrading strains [39]. While all the Colombian *A. ureafaciens* strains and the Indian strain were isolated from agricultural soils, 5 of the Chinese strains were reported to originate from the wastewater of atrazine plants. The other 3 Chinese *A. ureafaciens* strains were agricultural isolates originated from Shandong Province (*Arthrobacter* sp. SD41 [34]) as well as Jiangsu (*Arthrobacter* sp. ADH-2 [30]) and Hebei (*Arthrobacter* sp. DAT1 [33]) provinces bordering Shandong to the south and north-west, respectively. Thus, atrazine-degrading bacteria belonging to the species *A. ureafaciens* appeared to have intercontinental distribution and to be the most frequently isolated atrazine degraders in China.

Strains of ERIC type E D5rh3-2 and D6rh3-5 tightly clustered with the type strain of *Arthrobacter sulfonivorans*. Strains D5-8, D5-13 and D6-1 (ERIC type L) were included in the cluster of species *Arthrobacter scleromae*, *Arthrobacter oxydans* and *Arthrobacter polychromogenes.* Strain D5-6 (ERIC type M) was closely related to *Arthrobacter phenantrenivorans* type strain. Strain D5-11 (ERIC type N) clustered with the type strain of *Arthrobacter subterraneus*. These 4 robust clusters did not contain known atrazine-degraders.

A highly robust cluster comprised the type strain of *Arthrobacter crystallopoietes* with two tight sub-clusters of atrazine degraders. One of the sub-clusters contained the atrazine-degrading bacterium *Arthrobacter* sp. AG1 isolated from the heavily contaminated industrial soil in China [26] and single representatives of ERIC type I (strain D5-3) and ERIC type J (strain D5-5). The other sub-cluster joined strains D3-3, D3-6 (ERIC type H), and D5-7 (ERIC type K) with atrazine-degrading strain *A. crystallopoietes* Cit2 isolated from French agricultural soils [43].

Isolates of ERIC types C and D were included into 2 distant phylogenetic groups of the genus *Nocardioides* (Fig. 2b). Strains of the ERIC type C and atrazine-degrading strain *Nocardioides* sp. AN3, isolated from riverbed sediment in Japan [44], formed a distinct subgroup within a robust cluster comprising the type strains of *Nocardioides marinus*, *Nocardioides panacihumi*, *Nocardioides terrae*, and the known atrazine-degrading isolates *Nocardioides* sp. CMU1 and CMU2 which originated from the United States [45]. ERIC type D isolates from GD and WS soils fitted in the large robust cluster formed by type strains of the species *Nocardioides alpinus*, *Nocardioides furvisabuli*, *Nocardioides exalbidus*, *Nocardioides oleivorans*, *Nocardioides ganghwensis*, 3 atrazine-degrading *Nocardioides* isolates from Colombian agricultural soils [39] and the French strain *Nocardioides* sp. SP12 reported to inhabit both maize rhizosphere and bulk soil [38]. Within this cluster, ERIC type D isolates were close to the type strain of *N. ganghwensis*. To our knowledge, atrazine-degrading *Nocardioides* spp. had not been previously isolated from Chinese soils.

Isolate D3-8 (ERIC type F) tightly clustered with the type strain of *Gulosibacter molinativorax* (Fig. 2b). The species was described based on characterization of a single strain, capable of transforming the herbicide molinate [46]. To our knowledge, this is the first report of an atrazine-degrading strain belonging to the genus *Gulosibacter*, and to the family *Microbacteriaceae*.

Bacteria D3-1l and D3-2 clustered with the type strain of *Pseudomonas alcaliphila* (Fig. 2c). This cluster also contained known atrazine-degrading bacterium *Pseudomonas* sp. AD39 isolated from the wastewater system of atrazine plant in China [28]. There were no differences between nucleotide sequences of 16S rRNA genes from D3-1l, D3-2 and *P. alcaliphila* AL15-21^T^ in the final dataset of the alignment. However, the sub-cluster had medium level of the bootstrap support, being included in a robust cluster together with closely related type strains *Pseudomonas oleovorans* ssp. *lubricantis* RS1^T^, *Pseudomonas toyotomiensis* HT-3^T^ and *Pseudomonas pseudoalcaligenes* Stanier 63^T^.

Despite the method of direct plating enabled us to discover a variety of atrazine degraders, the diversity revealed was limited to the genera *Arthrobacter*, *Nocardioides*, *Gulosibacter* and *Pseudomonas*. The diversity of atrazine-degrading *Arthrobacter* spp. originated from three adjacent sites of industrial soils exceeded taxonomic diversity of known atrazine-degrading *Arthrobacter* bacteria isolated worldwide. At the same time, representatives of most other known atrazine-degrading genera, such as *Clavibacter*, *Nocardia* (phylum *Actinobacteria*), *Agrobacterium, Alcaligenes, Herbaspirillum, Pseudaminobacter, Pseudomonas, Polaromonas, Ralstonia, Rhizobium, Sinorhizobium, Stenotrophomonas* (phylum *Proteobacteria*) [15] were not found among the isolates, suggesting that they were either absent in the soils studied or were present as minor groups not directly detectable among the dominating atrazine degraders. Drastic differences between the genetic structures of the atrazine-degrading communities in industrially contaminated soils at different sites, and between those in the rhizosphere of plants growing in the industrially contaminated and agricultural soils clearly indicate the selection of bacteria that are better adapted to local environments. As a result, representatives of different species, genera, or even phyla became prevalent in the atrazine-degrading communities. However, in all sites except D3 where *Gulosibacter* strains were isolated, the adaptation did not extend the diversity of prevalent atrazine degraders over the range of known atrazine-degrading genera. Interestingly, that no *Pseudomonas* strains, considered to be excellent root-colonizing bacteria [47], or other atrazine degrading strains belonging to the genera of the phylum *Proteobacteria* [15], both culturable and unculturable representatives of which were proved to dominate in root-associated bacterial consortia [48, 49], were found among the rhizosphere isolates. This was especially notable for isolates from D5 and D6 sites where cogon grass and common reed thickets were close to the plantless D3 soil in which atrazine degrading bacteria of *P. alcaliphila* phylogenetic group were abundant. This fact indicates a failure of horizontal transfer of functional atrazine degradation genes to the rhizosphere-competent pseudomonads or other *Proteobacteria*. Alternatively, it demonstrates a low contribution of the associative plant-bacterial interaction [47, 50] to the rhizosphere competence of bacteria and their population growth compared to the advantages gained by *Arthrobacter* bacteria from the effective TrzN-mediated utilization of atrazine.

The observed limitation of atrazine degraders’ diversity to various *Arthrobacter* spp. and some phylogenetic groups within a narrow range of other genera suggests that individual atrazine utilization is an uncommon capability of soil bacteria belonging to a limited number of lineages. Probable reasons for this may be a lack of efficient gene transfer systems or a physiological incompatibility of potential recipients with functional atrazine degradation genes. However, the genes *atzABC* are highly mobile, and their intergeneric transfer in the rhizosphere has been demonstrated [51]. Although horizontal transfer of *trzN* still has not been documented, location of the gene within transposon-like structures, on plasmids, and its presence in viral DNA suggest that *trzN* can spread and facilitate the adaptation of microbial communities to contamination by atrazine [52]. The high local diversity of bacteria harboring *trzN* in industrial soils found in this study also implies ecological significance of *trzN* mobility. Thus, the latter assumption looks more likely. Full or partial incompatibility of the pathways for fast atrazine degradation with physiological background of a host may interfere with utilization of atrazine or reduce soil or rhizosphere competence of bacteria acquired the atrazine degradation genes.

### BOX-PCR genotyping of ERIC type B atrazine-degrading isolates

All related to *A. ureafaciens* atrazine-degrading isolates of ERIC type B exhibited identical ERIC-PCR patterns regardless their geographical origin. It was known that BOX-PCR genotyping (PCR targeting bacterial repetitive BOX element) revealed a higher diversity in Colombian atrazine-degrading strains of *A. ureafaciens* [39]. Aiming to examine their genetic uniformity, the ERIC type B isolates were genotyped by BOX-PCR. The type strain *A. ureafaciens* CGMCC 1.1897^T^ was included in the analysis in order to obtain more taxonomic information. Earlier, ERIC–PCR revealed no similarity between representative ERIC type B bacteria and *A. ureafaciens* type strain (Additional file 8: Figure S6). The amplicon patterns generated from BOX-PCR with DNAs of ERIC type B isolates and strain *A. ureafaciens* CGMCC 1.1897^T^ demonstrated marked identity (Additional file 9: Figure S7). Taking into account that Rep-PCR genotyping resolved genetic differences between strains within the same species [53], the BOX-PCR results provided evidence that ERIC type B atrazine degraders isolated in this work were a group of genetically similar bacteria belonging to the species *A. ureafaciens*.

### Detection of atrazine degraders in soils by conventional PCR targeting genes for atrazine degradation

A narrow taxonomic range of atrazine degraders isolated in this work might be a result of limited culturability of some bacteria. Also, the transformation of atrazine in soils can be carried out by bacterial consortia whose members separately contain the genes *trzN*, *atzA*, *atzB* and *atzC* [15]. In order to better understand the role of the isolated atrazine degraders in atrazine-degrading communities in the industrial and agricultural soils, we analyzed the soil DNAs by conventional PCR targeting the genes *trzN*, *atzA*, *atzB* and *atzC*.

Selectivity of the primers for atrazine degradation genes was previously assessed in gradient PCRs with S1 soil DNA as a template. It was found that although no non-target nucleotide sequences with substantial complimentarily to the designed primers were found in the GenBank databases, most of the tested primer pairs yielded multiple unintended products at T_a_ values close to or even higher than the calculated melting midpoints (Additional file 10: Figure S8). This suggested that annealing of these primers to partly complementary non-target templates might interfere with annealing to the perfect complements present in the soil DNA extracts at much lower concentrations, thus reducing the sensitivity of PCR detection. No amplification of unintended products was observed in PCRs with primer pairs *atzA*655f/*atzA*982r, *atzB*181f/*atzB*316r and *atzC*340f/*atzC*552r (Additional file 10: Figure S8) indicating a high specificity. Primer pair *trzN*1114f/*trzN*1271r produced unintended products at T_a_ range from 58 to 64 °C, but the visible yield was much lower than that detected in PCR with the commonly used pair C190-10/C190-11.

**Fig. 3:**
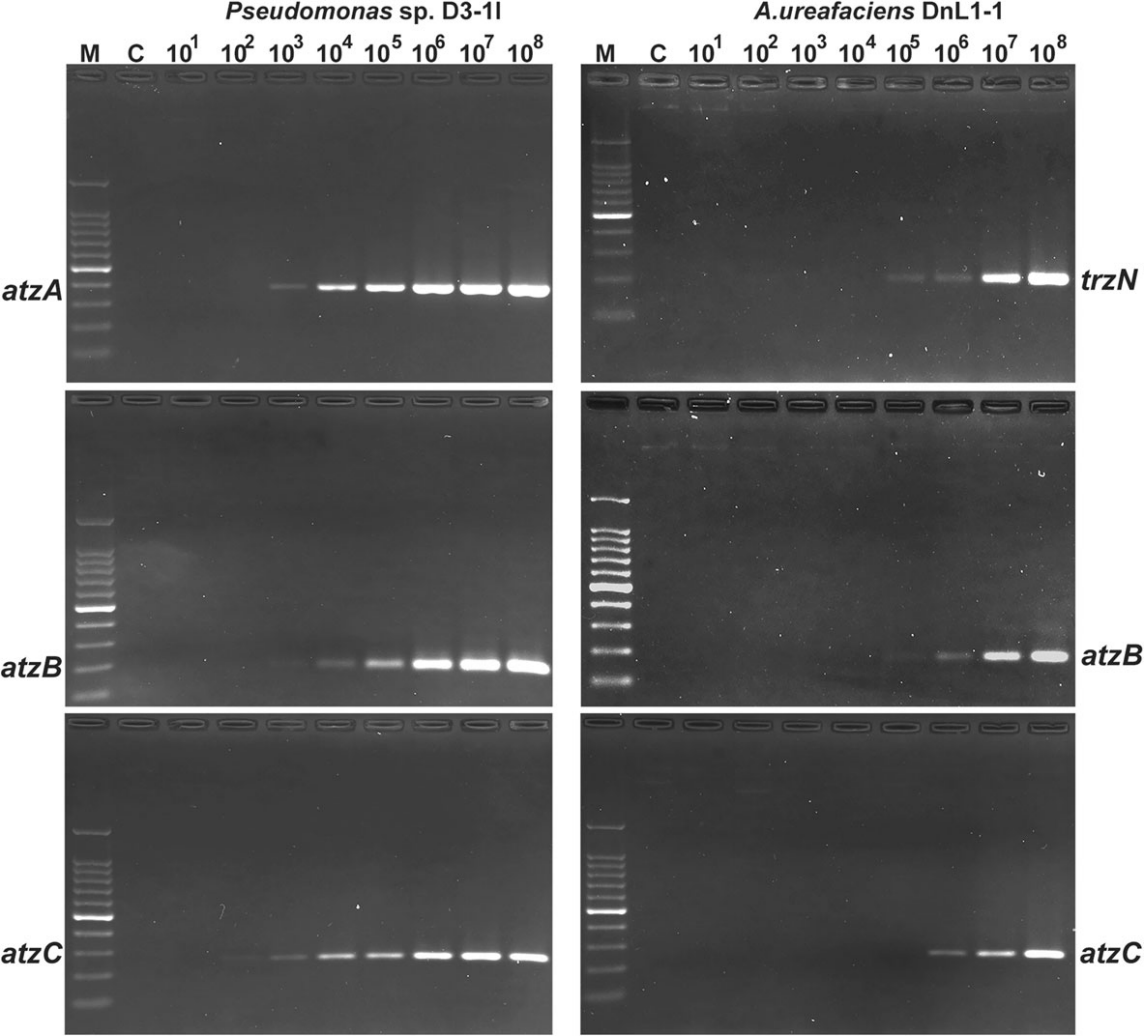
The limits of *Pseudomonas* sp. D3-1l and *A. ureafaciens* DnL1-1 detection in soil by PCRs targeting atrazine degradation genes. Lanes are designated by titers of *Pseudomonas* sp.D3-1l (*left panel*) and *A. ureafaciens* DnL1-1 (*right panel*) CFU added per 1 g of S1 soil. Lane N and C are, respectively, no template control and a control in which DNA isolated from non-inoculated S1 soil was used as a template. The primer pairs for *trzN*, *atzA*, *atzB* and *atzC* were *trzN*1114f/*trzN*1271r, *atzA*655f/*atzA*982r, *atzB*181f/*atzB*316r and *atzC*340f/*atzC*552r, respectively. Lanes M contain a 100 bp DNA Ladder (Takara Biotechnology (Dalian) Co., Ltd., China)

To evaluate the detection limits of PCRs with the selected primer pairs, aliquots of S1 soil were spiked with known titers of isolates *Pseudomonas* sp.D3-1l or *A. ureafaciens* DnL1-1 prior DNA extraction. Conventional PCRs with the selected primer pairs *atzA*655f/*atzA*982r and *atzB*181f/*atzB*316r and template DNA extracted from S1 soil supplemented with at least 10^3^ CFU g^−1^ of *Pseudomonas* sp.D3-1l yielded products of the expected sizes near 0.37 kb and 0.18 kb, respectively (Fig. 3), demonstrating robust detection of this strain. Sensitivity of the PCR assay with primers *atzC*340f/*atzC*552r was even higher and a slight band of the expected 0.25 kb product indicated the detection of *Pseudomonas* sp.D3-1l population even at a density as low as 10^2^ CFU g^−1^ soil. No amplification was detected with DNAs isolated from S1 soil to which *Pseudomonas* sp.D3-1l was not added or its density was nearly 10^1^ CFU g^−1^ soil. In contrast to the strain *Pseudomonas* sp.D3-1l, the limits of *A. ureafaciens* DnL1-1 detection by PCR with primer pairs *trzN*1114f/*trzN*1271r and *atzB*181f/*atzB*316r were at the level of 10^5^ CFU g^−1^ soil, and nearly 10^6^ CFU g^−1^ soil with the pair *atzC*340f/*atzC*552r (Fig. 3). The band intensity in reactions with either of the primer pairs increased with CFU density, indicating that the assays could be used semi-quantitatively.

The fragments produced in PCRs with DNA extracted from S1 soil harboring *Pseudomonas* sp. D3-1l or *A. ureafaciens* DnL1-1 were sequenced to verify their identity to the targeted genes. The nucleotide sequences of the recovered *atzA* and *atzC* fragments were deposited in GenBank (Accession Nos. KP997248 - KP997250). The nucleotide sequences of short *trzN* fragment from *A. ureafaciens* DnL1-1 and *atzB* fragments from both strains are provided in Additional file 6. BLAST search results gave evidence that all the recovered fragments of the atrazine degradation genes shared 100 % identity with respective genes from known atrazine degrading bacteria, including *atzA*, −*B* and –*C* genes from *Pseudomonas* sp. ADP and *trzN*, *atzB* and –*C* genes from *A. aurescens* TC1.

A drastic difference between the limits of *Pseudomonas* sp.D3-1l and *A. ureafaciens* DnL1-1 detection can be explained by low extractability of DNA from the latter strain, rather than by actual selectivity and sensitivity of the reactions. A bias produced by DNA extraction method is known to affect composition and abundance of bacterial phylotypes which can be detected in soil [54, 55]. Feinstein et al. [56] found that the phylum *Actinobacteria* fell into the poorly lysed portion of soil bacterial communities. Our results demonstrate a possible link between the extraction bias and properties of the particular strains. Because *A. ureafaciens* bacteria appear to be predominant atrazine degraders in agricultural soils, the observed detection limit of 10^5^ CFU g^−1^ soil can result in underestimation of the atrazine-degrading populations by culture-independent methods or even cause false negative results.

The attained sensitivity of *Pseudomonas* sp.D3-1l detection in soil significantly exceeded those of previously described assays exploiting conventional PCR. Thus, the reported detection limits of *E. coli* strain harboring *atzA* or *trzN* cloned in a high copy number plasmid pCR 2.1-TOPO (Invitrogen, USA) targeted by the commonly used primer pairs *atzA*f/*atzA*r [21] or C190-10/C190-11 [22] respectively were at the level of 10^4^ CFU g^−1^ soil [39]. The same *atzA*f/*atzA*r primers allowed detecting of *Pseudomonas* sp. ADP at its density no less than 10^6^ CFU g^−1^ soil, and additional purification or dilution of soil DNA did not improve the sensitivity of the assay [57]. The reported detection limit for *Nocardioides* sp.C190 targeted by primers C190-10/C190-11 was 10^8^ CFU g^−1^ soil [22]. As a result, detection of atrazine degradation genes in soil DNA by conventional PCR required prior “microcosm enrichment” of atrazine degraders [39].

The experiments with strain *Pseudomonas* sp.D3-1l provided evidence that conventional PCR exploiting commercial high-yield *TaKaRa Ex Taq* polymerase kit and primers designed in this work is sensitive enough to detect atrazine degraders in the 10^3^ CFU g^−1^ spiked soil sample. However, like methods of cultivation, direct PCR-detection of bacteria in soil has its own specific limitations, most likely caused by a poor lysability of many bacteria [56]. Thus, a combination of culturing and culture-independent methods seems to be a reasonable approach to extend the range of detectable atrazine degraders and to improve the detection sensitivity.

**Fig 4:**
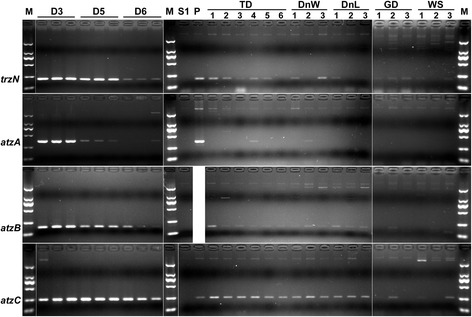
Detection of atrazine-degrading bacteria in soils by PCRs targeting the genes *trzN*, *atzA*, *atzB* and *atzC*. Lanes are designated by names of the sampling sites listed in Table 1. Numbers of replicate samples are given below the designations of sampling sites. Site TD(a) was represented by replicate samples 1–3, and site TD(b) by replicate samples 4–6. The primers for *trzN*, *atzA*, *atzB* and *atzC* were *trzN*1114f/*trzN*1271r, *atzA*655f/*atzA*982r, *atzB*181f/*atzB*316r and *atzC*340f/*atzC*552r, respectively. Lanes P contain products of positive control reactions for which template DNAs were isolated from S1 soil supplemented with 10^4^ CFU g^−1^ of *Pseudomonas* sp.D3-1l for *atzA* gene and with 10^6^ CFU g^−1^ of *A. ureafaciens* DnL1-1 for *trzN* and *atzC*. Positive controls for the gene *atzB* were reactions with template DNAs isolated from S1 soil aliquots supplemented with known titers of *Pseudomonas* sp.D3-1l (Additional file 11: Figure S9). Lanes M contain DL 2000 DNA Marker (Takara Biotechnology (Dalian) Co., Ltd., China)

The analysis of DNAs extracted from bulk D3 and D5 soils clearly identified the genes *trzN*, *atzA*, *atzB*, and *atzC* (Fig. 4). No products of the expected sizes were amplified in reactions with S1 soil DNA that served “no template control”. Reactions with D3 soil DNAs produced equally strong amplicon bands for all the targeted genes. At the same time, assays for the gene *atzA* with D5 template gave bands of slight intensity, indicating a lower density of *atzA*-harboring bacteria. PCRs with D6 soil DNAs clearly demonstrated amplification of the fragments for the genes *trzN*, *atzB*, and *atzC*, while a slight fragment of the expected size for the gene *atzA* was produced in reactions with only 1 of the 3 independent replicate templates.

In agricultural soils, the gene *atzA* was detected only in replicate samples TD4 and DnW2. The genes *trzN*, *atzB* and *atzC* were found in the maize rhizosphere at all agricultural sampling sites (Fig. 4). However, the gene *trzN* was not detected in some of TD(b), DnL, DnW and WS replicate samples, and the genes *atzB* and *atzC* in some of GD and WS replicate samples.

The results of the PCR assays matched the results of the direct isolation of atrazine-degrading bacteria. Both methods demonstrated the striking prevalence of atrazine degraders possessing the gene *trzN* in all industrial and agricultural soils studied, except D3 soil where culturable *atzA*-bearing pseudomonads were one of the major groups in the atrazine-degrading community.

Weak amplicon bands produced in positive reactions and negative results obtained for some replicate samples indicated that the population size of atrazine degraders in agricultural soils was close to the limit of their detection by RCR. Direct plating on SM agar revealed *A. ureafaciens* bacteria of ERIC type B as a predominant group of atrazine degraders in all replicate samples from all agricultural sites tested. Population densities of culturable atrazine degraders exceeded the level of 10^4^ CFU g^−1^ soil only at sites TD(a) and GD (Table 2), that was below the limit of PCR detection for *A. ureafaciens* DnL1-1. However, less than 40 % of viable *A. ureafaciens* cells could be recovered on SM agar at this population density (see Methods section), indicating that actual titers of the atrazine degrader in the maize rhizosphere were significantly higher than those detected on SM plates. Adjustment based on the percent recovery gave the densities of atrazine-degrading *A. ureafaciens* in TD(a) and GD soils close to the limit of their detection by PCR assay (10^5^ CFU g^−1^ soil), suggesting that *A. ureafaciens* bacteria might represent a substantial or even dominant fraction of atrazine-degrading communities in agricultural soils.

## Conclusions

The present study provides the first example of direct detection and isolation of atrazine-degrading bacteria on a specially developed selective agar SM with atrazine as sole nitrogen source and Tween 80 as wetting agent and dispersant. This method overcomes the limitations of common enrichment protocols and facilitates enumeration and rapid isolation of atrazine degraders. Elimination of the enrichment bias enables analysis of the diversity and community structure of culturable atrazine degrading bacteria in soil. Advantages of the direct isolation make it a reasonable method for future investigations of the ecology and biogeography of atrazine degraders.

The highly specific primers designed in this work enabled PCR-detection of atrazine-degrading *Pseudomonas* sp. populations as low as 10^3^ CFU g^−1^ soil. However, the assay was at least 100 times less sensitive for the detection of atrazine-degrading *A. ureafaciens*, indicating that its results could be greatly affected by the differential DNA extractability from targeted soil bacteria. For this reason, the PCR assay is a useful supplemental method of the detection rather than an alternative to the direct plating.

Both the direct plating and culture-independent assays provided evidence that the atrazine degraders constituted a major component of microbial populations in industrially contaminated soils and a minor one in the maize rhizosphere. The industrial soils harbored communities of genetically distinct bacteria that were individually capable of degrading and utilizing atrazine. Genetic structures of the atrazine-degrading communities in soils differentially exposed to atrazine did not overlap, indicating intensive selection of bacteria better adapted to local environments. However, the range of the atrazine degraders was limited to a variety of phylotypes belonging to the genus *Arthrobacter*, 2 phylotypes within the genus *Nocardioides*, and single genotypes within phylogenetic groups of *Pseudomonas alcaliphila* and *Gulosibacter molinativorax*, suggesting that the individual atrazine utilization is a trait of soil bacteria belonging to a limited number of lineages.

Strains of *P. alcaliphila* phylogenetic group were the only harboring the gene *atzA*, while all other isolates possessed *trzN*. The strong prevalence of *trzN*-bearing atrazine degraders in all the industrial and agricultural soils, except that from the site where atrazine-degrading pseudomonads were isolated, was confirmed by PCR assay. Bacteria related to *P. alcaliphila*, *G. molinativorax* and *A. crystallopoietes* were major atrazine-degrading inhabitants of the heavily contaminated plantless soil. The rhizosphere of growing in the industrial soils cogon grass and common reed harbored an abundance of atrazine-degrading *Arthrobacter* spp. with a strong prevalence of a genomospecies closely related to, but distinct from, the species *A. aurescens*, *A. ilicis*, and *A. nitroguajacolicus*.

In contrast to the diversity of atrazine-degrading *Arthrobacter* spp. in industrial soils, genetically similar *A. ureafaciens* bacteria were the dominant culturable atrazine degraders and the only found atrazine degrading representatives of the genus in the maize rhizosphere at all agricultural sampling sites. The contribution of *A. ureafaciens* bacteria to the enhanced degradation of atrazine in agricultural soils and mechanisms causing their prevalence among atrazine degraders in the maize rhizosphere deserve further study.

## Methods

### Soil sampling and processing

The geographic coordinates of sampling sites were determined using GPS + GLONASS receiver Garmin eTrex 30 with an accuracy of 3 m. Cropping and herbicide histories for at least the past 5 years were obtained from specialists of the local agriculture bureaus and farm managers. At each field site, 3 replicate samples were collected 2.0 – 2.5 m apart and then processed independently. Soil cores 15 × 10 × 10 cm (length × width × depth) containing grass roots or the root system of an individual maize plant were carefully excavated with a surface-sterilized shovel avoiding mechanical disturbance to the samples in order to retain intact soil structure. Shoots of common reed and cogon grass were cut to a length of 15 cm, and stems of the maize plants were cut above the first node. The intact soil cores were placed into plastic boxes, delivered to the laboratory the day of sampling and kept at room temperature (23–25 °C) overnight till processing.

Soil samples were processed aseptically to prevent cross-contamination. S1 soil was sieved to 2 mm and thoroughly mixed. Triplicate 20 g sub-samples of the homogenized S1 soil were oven-dried at 105 °C to constant weight in order to determine moisture content.

For isolation of atrazine degraders, soil samples were processed the day after delivery (within 22–26 h after sampling). Plant roots were carefully retrieved from the cores and the loose soil was removed by hand using sterile latex gloves. Roots of common reed and cogon grass with tightly adhering soil were cut from the rhizomes, clipped into approximately 2.5 cm pieces, mixed in a Petri dish and then directly used in the isolation procedure. To recover rhizosphere soil of maize, the soil tightly associated with seminal and nodal roots was scraped off with the blunt side of a scalpel, placed into a Petri dish and mixed. Soil remaining in the box was considered to be bulk soil. The bulk soils were homogenized and the moisture contents were determined in the manner described for S1 soil. Because the high humidity prevented sieving the agricultural bulk soils, they were preliminary air-dried for 2 days. For this reason, dry matter contents required for normalizing CFU densities of detected atrazine-degraders were determined in 20 g sub-samples of agricultural bulk soils collected before their drying and further processing.

Samples of bulk soils reserved for analysis of texture and chemical characteristics were kept at −20 °C. Soil characteristics were analyzed in the Laboratory of Environmental Analysis of the Shandong Provincial Analysis and Test Center.

### Atrazine extraction and quantification

Extraction of atrazine from soil samples was performed in the manner described by Krutz et al. [17]. The extracts were analyzed by high performance liquid chromatography-tandem mass spectrometry (HPLC-MS/MS) on an UltiMate 3000 HPLC system interfaced to a TSQ Vantage Triple Quadrupole Mass Spectrometer (Thermo Fisher Scientific, USA). The conditions of HPLC and MS/MS parameters are provided in Additional file 12. Calibration standards of atrazine (# 45330 Sigma-Aldrich, USA) were prepared in HPLC grade methanol (Sinopharm, China) at the following concentrations: 0.01, 0.05, 0.1, 0.5, 1, 5, 10, 50 and 100 ng mL^−1^. The typical calibration curve was Y = 2313.0 + 4073.8X. The curve displayed excellent linearity (r^2^ = 0.9995) over the entire calibration range. Recovery of atrazine from fortified soil samples was 90.3–93.2 %. The method limit of atrazine quantification was 0.05 μg kg^−1^.

### Bacterial strains

The strain *Arthrobacter* sp. SD41 was previously isolated from the rhizosphere of wheat sampled in Yucheng County, Dezhou Prefecture of Shandong Province [34]. The species type strain *A. ureafaciens* CGMCC 1.1897^T^ was obtained from the China General Microbiological Culture Collection Center (www.cgmcc.net).

### Growth media

Mineral media SM, SMY and nutrient medium TY were used for enumeration, isolation, purification and maintenance of atrazine-degrading bacteria according to the methods described. The medium SM contained (per liter of distilled water) 0.5 g K_2_HPO_4_, 0.2 g MgSO_4_ · 7H_2_O, 0.1 g NaCl, 0.02 g CaCl_2_, 2 g D-glucose, 10 mL atrazine stock solution, 5 mL ZnFe-citrate stock solution. The atrazine stock solution contained (per 100 mL of distilled water) 1 mL Tween 80 and 5 g atrazine powder (≥97 %, Shandong Dehao, China). ZnFe-citrate stock solution contained (per 100 mL of distilled water) 0.04 g ZnSO_4_ · 7H_2_O, 0.4 g FeSO_4_ · 7H_2_O and 10 g trisodium citrate. SM25 medium was SM with concentration of atrazine reduced to 25 mg L^−1^. SMY medium was SM amended with 0.1 g L^−1^ yeast extract (Oxoid, England). TY medium contained (per liter of distilled water) 10 g tryptone (Oxoid, England), 1 g yeast extract (Oxoid, England), and 0.02 g CaCl_2_. The solid media SM, SMY and TY were supplemented with 13 g L^−1^ bacteriological agar (Beijing Dingguochangsheng Biotechnology Co., Ltd., China).

### Recovery of a reference atrazine-degrading strain

The recovery of atrazine degraders was studied after inoculation of S1 soil with known titers of *Arthrobacter* sp. SD41. Previously, the presence of atrazine degraders in S1 soil was checked by enrichment in liquid SM medium performed in the manner described by Mandelbaum et al. [35]. No visible dissolution of atrazine was observed in the cultures during 3 enrichment cycles, and no colonies with clearing zones were detected after their plating on SM agar, indicating no culturable atrazine degraders.

To prepare inocula, twenty 3 day-old colonies of the strain *Arthrobacter* sp. SD41 were harvested from an SMY plate, suspended in 1 mL buffer (SM medium salt solution), twice washed by centrifugation (3000 × g, 2 min) and serially diluted. CFU titers were determined by plating the dilutions on SM agar and incubation at 28 °C for 4 days.

Raw S1 soil was weight (100 mg dry weight equivalents) to 2 mL polypropylene tubes. The samples were spiked with the dilutions of *Arthrobacter* sp. SD41 cell suspension (20 μL/ sample), incubated on the bench for 5 min., and then 1 mL buffer was added to each tube. The tubes were secured in a MO BIO Vortex Adapter assembled on a Vortex-Genie® 2 Vortex (MO BIO Laboratories, Inc., USA) and vortexed at maximum speed for 10 min. The resulting soil suspensions and their serial dilutions were plated on SM agar. Colonies with typical zones of atrazine dissolution were counted after 4-day incubation at 28 °C. Mean CFU numbers, percent recoveries and 95 % confidence intervals were calculated based on 3 replications for each dilution by using the descriptive statistics tool of MS Excel.

Direct plating of the inoculated S1 soil on SM agar enabled selective isolation of *Arthrobacter* sp. SD41 (Additional file 13: Figure S10). Nearly 1/3 of the bacterial cells were recovered at inoculation densities from 10^3^ to 10^5^ CFU g^−1^ soil (Table 5). No atrazine-degrading colonies were recovered from S1 soil spiked with 10^2^ CFU g^−1^, indicating the method limit of *Arthrobacter* sp. SD41 detection was 10^3^ CFU g^−1^ soil. Values close to full recovery were obtained at inoculation density near 10^6^ CFU g^−1^ soil, or about 1 % of the resident population, (1.2 ± 0.1) × 10^8^ CFU g^−1^ soil, enumerated by plating on TY agar. Recoverability of *Arthrobacter* sp. SD41 from soil dilutions directly plated on SM agar was similar to that obtained for Tn*5*-marked *Pseudomonas putida* and *Rhizobium* spp. enumerated by the most-probable-number–DNA hybridization procedure [58]. Hence, the direct plating on SM agar was considered a promising method of detection, enumeration and isolation of atrazine-degrading bacteria from industrially contaminated and agricultural soils.

**Table 5:** Recovery of *Arthrobacter* sp.SD41 from S1 soil by direct plating on SM agar

Inoculation density, CFU g^−1^ soil	Recovery, CFU g^−1^ soil^a^	Percent recovery^a^
No inoculation (control)	˂10^2^	ND
1.6 × 10^2^	˂10^2^	ND
1.6 × 10^3^	(6.0 ± 2.4) × 10^2^	37.5 ± 15.0
1.6 × 10^4^	(5.5 ± 0.7) × 10^3^	34.8 ± 4.8
1.6 × 10^5^	(6.3 ± 0.8) × 10^4^	39.4 ± 5.0
1.6 × 10^6^	(1.4 ± 0.1) × 10^6^	87.5 ± 6.3

^a^ Means and 95 % confidence intervals. *ND* not determined

### Detection, enumeration and isolation of atrazine-degrading bacteria

Isolation of atrazine-degrading bacteria was commenced immediately after the processing of soil samples. To detect, enumerate and isolate atrazine degraders, 0.1 g of bulk or rhizosphere soil, or (for samples D5 and D6) 0.1 g of common reed or cogon grass root sections with tightly adhering soil were placed into 2 mL polypropylene tubes with 1 mL washing buffer (SM medium salt solution). The tubes were secured in a MO BIO Vortex Adapter assembled on a Vortex-Genie® 2 Vortex (MO BIO Laboratories, Inc., USA) and vortexed at maximum speed for 10 min. Washed root sections from D5 and D6 rhizosphere samples were removed, blotted and weighed to determine the exact amount of soil in the tubes. Serial dilutions of the resulting suspensions were plated onto solid media SM and TY. The plates were incubated at 28 °C. Colonies of soil bacteria growing on TY agar were counted after 5 days. Colonies of atrazine degraders, which produced clearing zones, were first counted after 3-day incubation. The appearance of additional atrazine-degrading colonies was checked daily during the following week. Bacterial densities and confidence intervals were calculated according to Koch [59].

For isolation of atrazine degraders, colonies were chosen based on their morphology, size of clearing zones and time required for their production. Several colonies within each distinguishable type were selected to ensure the most complete isolation of different bacteria. The colonies were repeatedly streaked on SMY agar until cultures of atrazine-degrading bacteria showing no presence of contaminating microorganisms on SMY, TY and R2A (Sigma-Aldrich, USA) agar media were obtained.

### Evaluation of atrazine-degrading capacity of isolates

Strains of atrazine degraders were cultured in 25 mL of SM25 medium in 250 mL Erlenmeyer flasks without shaking at 28 °C for 7 days. After incubation, the cultures were centrifuged (4000 g, 2 min.). Clear cultural liquids were transferred to polypropylene tubes and kept at −20 °C until analysis. The medium SM25 kept at −20 °C from the beginning of the experiment, and the medium SM25 incubated under the same conditions as the cultures were used as controls. The liquids were analyzed by HPLC-MS/MS in the above described manner.

### DNA extraction

Template DNAs for PCR were extracted from pure cultures of the isolates grown on SMY agar by thermal lysis in a 5 % suspension of Chelex 100 resin (BioRad, USA) performed in the manner described by Mahenthiralingam et al. [60].

Soil DNA was extracted by using a Power Soil DNA Isolation Kit (MO BIO Laboratories, Inc., USA), according to the manufacturer’s instructions, immediately after processing of the soil samples. The isolated soil DNAs were kept at −70 °C for analysis.

### PCR primers

Characteristics of primers used in this research are summarized in Table 6. All primers were synthesized by Sangon Biotech (Shanghai) Co., Ltd., China.

**Table 6:** DNA primers

Target gene	Primer^a^	Nucleotide sequences (5' → 3')	Reference
*atzA*	*atzA*250f	TCGCACGGGCGTCAAT	This study
	*atzA*655f	CGCTCCTGCCACTACCA	This study
	*atz*A650r	TGTCACCGCCGTGGTAG	This study
	*atz*A757r	GCGGGACTCATCCCATGAAT	This study
	*atzA*982r	TACGGAGTCATTACTATTCCCGTT	This study
*atzB*	B1f	AGGGTGTTGAGGTGGTGAAC	[51]
	B1r	CACCACTGTGCTGTGGTAGA	[51]
	*atzB*181f	GGGTGTTGAGGTGGTGAACT	This study
	*atzB*426f	ACCAGTACAACTACAGCCGC	This study
	*atzB*681f	TGATTGCCTACCCGGAAACC	This study
	*atzB*316r	TCTTCATCCACCAGGGCAAA	This study
	*atzB*662r	GGTTTCCGGGTAGGCAATCA	This study
	*atzB*919r	CTTCGGCACCCACCAGAAA	This study
*atzC*	*atzC*f	GCTCACATGCAGGTACTCCA	[21]
	*atzC*r	TGTACCATATCACCGTTGCCA	[21]
	Cf	GCTCACATGCAGGTACTCCA	[51]
	C1r	TCCCCCAACTAAATCACAGC	[51]
	*atzC*340f	TGTGATAGAACATGCTCACATGC	This study
	*atzC*552r	TAGCAGGATCAACTCCCCCA	This study
*trzN*	C190-10	CACCAGCACCTGTACGAAGG	[22]
	C190-11	GATTCGAACCATTCCAAACG	[22]
	*trzN*1114f	AATGGCAACCAGGGGATCAG	This study
	*trzN*1271f	GAGCACCTGACCATTCACGA	This study
*rrs*	63KWf	CAKGCCTWACACATGCAAGTC	[62], this study
	1389r	ACGGGCGGTGTGTACAAG	[62], this study
ERIC	ERIC2	AAGTAAGTGACTGGGGTGAGCG	[64]
BOX	BOXA1R	CTACGGCAAGGCGACGCTGACG	[65]

^a^Numbers in designations of the primers designed in this work are positions of their 3' nucleotides in coding direct sequences of the respective genes: *atzA*, *atzB*, *atzC* from *Pseudomonas* sp. ADP (GenBank accession no. U66917, regions 34964-36388, 44487-45932, 70219-71430 respectively), and *trzN* from *Nocardioides* sp. C190 (GenBank accession no. AF416746); f – forward, r - reverse

New primers were designed by using the NCBI primer-BLAST tool, ensuring verification of their specificity to the target genes *atzABC* from *Pseudomonas* sp. ADP (GenBank accession no. U66917, regions 34964–36388, 44487–45932, and 70219–71430 respectively), *atzABC* from *Herbaspirillum huttiense* B601 (GenBank accession nos. DQ089655, AY965854, and AY965855 respectively), *trzN* from *Nocardioides* sp. C190 (GenBank accession no. AF416746, region 4490–5860). Since high sequence similarity was found between the melamine deaminase gene *triA* from *Pseudomonas* sp. strain NRRL B-12227 and known *atzA* genes [61], the *atzA*-targeting primers were designed so as to match *atzA*-specific positions by their 3' nucleotides.

To amplify 16S rRNA gene sequences of the diverse bacteria, the primer pair 63KWf/1389r was designed, based on the primers 63f and 1387r, described by Marchesi et al. [62] as useful ones for ecological and systematic studies. Alignment with 16S rRNA gene sequences from the GenBank database revealed G or T in the 3^rd^ position, and A or T in the 8^th^ position from the 5′ end of the forward primer in many bacteria. Hence, these residues were synthesized as K and W respectively in the modified primer 63KWf. The 2^nd^ position from the 3′ end of the primer 1387r was also found to vary. Therefore, the primer 1389r was proposed instead of 1387r.

### PCR-detection and sequencing of atrazine degradation genes

All PCRs were done in a BioRad Verity thermal cycler.

Specificity of the primers targeting genes for atrazine degradation was assessed in gradient PCR with DNA isolated from S1 soil as a template. Reactions were performed in a total volume of 20 μL containing: 2 μL of *TaKaRa* 10× *Ex Taq* Buffer, (Takara Biotechnology (Dalian) Co., Ltd., China), 2.0 mM MgCl_2_, 200 μM of each dNTP, 0.5 μM of each primer, 0.5 U of *TaKaRa Ex Taq* polymerase (Takara Biotechnology (Dalian) Co., Ltd., China), and 0.5 μL of the soil DNA as a template. The temperature program was as follows: denaturation at 95 °C for 3 min; then 40 cycles consisting of 94 °C for 1 min, T_a_ range 58-68 °C with 2 °C step for 1 min, 72 °C for 1 min; and a final extension at 72 °C for 2 min. Conventional PCR to detect atrazine degradation genes in soil was performed in the same manner using hot start *TaKaRa Ex Taq* HS polymerase with the selected primer pairs *atzA*655f/*atzA*982r, *atzB*181f/*atzB*316r, *atzC*340f/*atzC*552r and *trzN*1114f/*trzN*1271r at T_a_ 68 °C. The number of cycles was reduced to 35.

Detection of the genes *atzA*, *atzB*, *atzC* and *trzN* in the isolated bacterial cultures was carried out by multiplex PCR using primer pairs *atzA*250f/*atzA*650r, *atzB*426f/*atzB*662r, *atzC*f/*atzC*r, and *trzN*1114/*trzN*1271r. Reactions were done in a total reaction volume of 20 μL containing: 2 μL of *TaKaRa* 10× *Ex Taq* Buffer, (Takara Biotechnology (Dalian) Co., Ltd., China), 2.0 mM MgCl_2_, 250 μM of each dNTP, 0.2 μM of each primer, 0.5 U of *TaKaRa Ex Taq* HS polymerase (Takara Biotechnology (Dalian) Co., Ltd., China) and 0.5 μL of a bacterial lysate as a template. The temperature program was as follows: denaturation at 95 °C for 3 min; then 25 cycles consisting of 94 °C for 1 min, 62° for 30 sec., 72 °C for 1 min; and a final extension at 72 °C for 2 min.

Fragments of the predicted sizes produced by the reference strains in the multiplex PCRs were cut out of the gel and purified using a *TaKaRa* MiniBEST Agarose Gel DNA Extraction Kit Ver. 4.0 (Takara Biotechnology (Dalian) Co., Ltd., China). Additionally, a 0.42 kb fragment of *trzN* from *Arthrobacter* sp. SD41 was amplified using a conventional primer pair C190-10/C190-11. The fragments were directly sequenced in both directions exploiting respective amplification primers. The sequencing reactions were performed using a BigDye Terminator v.3.1 Cycle Sequencing Kit (Applied Biosystems, United States) according to the manufacturer’s recommendations. Automated sequencing was performed on a 3730xl DNA Analyzer (Applied Biosystems, United States) at the Sequencing Department of the Sangon Biotech (Shanghai) Co., Ltd. The resulting DNA traces and sequences were checked and corrected manually.

Sequencing of the fragments produced in the PCRs targeting the genes for atrazine degradation in soil DNA was carried out in a similar manner.

### Genotyping

Genotyping of the isolates was performed by repetitive elements sequence-based PCR (Rep-PCR) with primers ERIC2 or BOXA1R (Table 6). The reaction mixture (20 μL) contained 2 μL of *TaKaRa* 10× *Ex Taq* Buffer, (Takara Biotechnology (Dalian) Co., Ltd., China), 2.0 mM MgCl_2_, 250 μM of each dNTP, 1.0 μM of one of the primers, 0.5 U of *TaKaRa Ex Taq* polymerase (Takara Biotechnology (Dalian) Co., Ltd., China) and 0.5 μL of a bacterial lysate as a template. Rep-PCRs were started by denaturation at 95 °C for 3 min. followed by 4 cycles at 94 °C for 1 min., 40 °C (ERIC-PCR) or 55 °C (BOX-PCR) for 1 min., 68 °C for 8 min.; followed by 30 cycles: 94 °C for 1 min., 52 °C (ERIC-PCR) or 65 °C (BOX-PCR) for 1 min., 72 °C for 2 min. A final extension was performed at 72 °C for 5 min.

Products of the amplification were separated by electrophoresis on a 2.0 % agarose gel (Genview, China) in 0.5 × TBE. The gel was supplemented with 50 μL L^−1^ GoldView Nucleic Acid Stain (Beijing Dingguochangsheng Biotechnology Co., Ltd., China) in order to visualize DNA bands. The Rep-PCR banding patterns were analyzed visually, and similar ones were considered to belong to the same genotypic group.

### Sequencing of 16S rRNA genes and phylogenetic analysis

The genes for 16S rRNA were amplified with the primer pair 63KWf/1387r. The PCRs were performed in a total volume of 50 μL containing 5 μL of *TaKaRa* 10× *Ex Taq* Buffer (Takara Biotechnology (Dalian) Co., Ltd., China), 2.0 mM MgCl_2_, 250 μM of each dNTP, 0.5 μM of each primer, 0.5 U of *TaKaRa Ex Taq* polymerase (Takara Biotechnology (Dalian) Co., Ltd., China) and 1.25 μL of a bacterial lysate as a template. The PCRs were started by denaturation at 95 °C for 3 min.; and consisted of 30 cycles: 94 °C for 1 min., 55 °C for 1 min., 72 °C for 2 min.; followed by extension at 72 °C for 5 min. The amplification products were analyzed by electrophoresis on an agarose gel in 0.5 × TBE. Target fragments of about 1.3 kb were cut out from the gel, purified and sequenced in the manner described for the fragments of atrazine degradation genes.

The BLASTn similarity search was performed against 16S ribosomal RNA sequences database of GenBank. The phylogenetic analysis was performed using the MEGA5 software package [63]. 16S rRNA gene nucleotide sequences of known atrazine-degrading bacteria and species type strains sharing more than 98 % sequence similarity with the analyzed bacteria were included in the datasets. Multiple alignments were implemented using the CLUSTALW aligner of MEGA5 and then refined by hand. Phylogenies were inferred using the Neighbor-Joining algorithm with elimination of all positions containing gaps and missing data.

## Additional files

Additional file 1: Figure S1. Map of Shandong Province and location of sampling sites. Location of sampling sites is indicated by the symbol ● with following designations: S - Jinan, Eastern Campus of the Shandong Academy of Sciences; D - Dajiawa Town, Weifang Prefecture; TD - Dongdaguan Village, Dawenkou Township, Tai’an Prefecture; DnW - Wangdian Village, Binhe Township, Dingtao County, Heze Prefecture; DnL - Liulou Village, Binhe Township, Dingtao County, Heze Prefecture; GD - Dawang Village, Guhe Township, Gaotang County, Liaocheng Prefecture; WS - Shadong Village, Guangyun Township, Wucheng County, Dezhou Prefecture. (TIF 1030 kb)
Additional file 2: Table S1. Soil characteristics. (DOC 50 kb)
Additional file 3: Figure S2. Direct isolation of atrazine-degrading bacteria on SM agar. 1^st^ dilution of soil suspension (a total of 5 mg soil) was spread on the medium surface. The total population of culturable bacteria was about 10^9^ CFU g^−1^ soil. The incubation time is indicated near the plates. (TIF 6889 kb)
Additional file 4: Figure S3. Discrimination of atrazine-degrading bacteria by ERIC-PCR banding patterns. ERIC types of the bacteria are designated by capital letters under the square brackets. Letters in the name of each strain are designations of the locations as indicated in Table 1. Numbers after letter D represent sampling sites at the location D as listed in Table 1. Letter “r” following D5 and D6 indicates the rhizosphere isolates. The first digit in the names of strains isolated from TD, DnW, DnL, GD and WS sites indicates replicate samples. Site TD(a) was represented by replicate samples 1–3, and site TD(b) by replicate samples 4–6. Lanes M contain DL 2000 DNA Marker (Takara Biotechnology (Dalian) Co., Ltd., China). (TIF 5554 kb)
Additional file 5: Figure S4. Amplification products obtained in multiplex gradient PCR targeting *trzN*, *atzA*, *atzB* and *atzC* with DNAs of the reference strains as templates. Lanes M contain DL 2000 DNA Marker (Takara Biotechnology (Dalian) Co., Ltd., China). (TIF 444 kb)
Additional file 6: Nucleotide sequences of short gene fragments. (TXT 1007 bytes)
Additional file 7: Figure S5. Detection of the genes for atrazine degradation in isolates by the multiplex PCR assay. Lanes are designated by the isolates’ names. Lanes M1 contain a 100 bp DNA Ladder, lane M2 - DL 2000 DNA Marker (Takara Biotechnology (Dalian) Co., Ltd., China). (TIF 4047 kb)
Additional file 8: Figure S6. ERIC-PCR patterns of representative ERIC type B isolates. Lanes are designated by the isolates’ names. Lane T – pattern of the species type strain *A. ureafaciens* CGMCC 1.1897^T^. Lane M contains DL 2000 DNA Marker (Takara Biotechnology (Dalian) Co., Ltd., China). (TIF 845 kb)
Additional file 9: Figure S7. BOX-PCR typing of ERIC type B isolates. Lanes are designated by the strain names. Lane T – pattern of the species type strain *A. ureafaciens* CGMCC 1.1897^T^. Lanes M contain a 100 bp DNA Ladder (Takara Biotechnology (Dalian) Co., Ltd., China). (TIF 1179 kb)
Additional file 10: Figure S8. Amplification of unintended products in PCRs with primers targeting the genes for atrazine degradation. Lanes are designated by values of T_a_. Lanes designated by M contain DL 2000 DNA Marker (Takara Biotechnology (Dalian) Co., Ltd., China). The primer pairs are printed on respective gel zones under or below banding patterns. (TIF 2535 kb)
Additional file 11: Figure S9. Detection of the gene *atzB* in PCRs with template DNAs isolated from S1 soil aliquots with known titers of *Pseudomonas* sp.D3-1l. (Positive controls for *atzB* detection results represented in Fig. 4). Lanes are designated by titers of *Pseudomonas* sp.D3-1l. Lane N and C are, respectively, no template control and a control in which DNA isolated from non-inoculated S1 soil was used as a template. Lanes M contain DL 2000 DNA Marker (Takara Biotechnology (Dalian) Co., Ltd., China). (TIF 385 kb)
Additional file 12: Conditions of HPLC and parameters of MS/MS. (DOC 76 kb)
Additional file 13: Figure S10. Recovery of *Arthrobacter* sp. SD41 from soil by direct plating on SM agar. S1 soil sample was inoculated with about 10^4^ CFU g^−1^ of *Arthrobacter* sp.SD41. 1^st^ (left dish) and 2^nd^ (right dish) dilutions of soil suspension were plated on SM agar. Photos of the dishes were taken after 3 (left) and 5 (right) days of incubation at 28 °C. (TIF 5262 kb)

## Abbreviations

BOX-PCR: Polymerase chain reaction targeting bacterial repetitive BOX element
ERIC-PCR: Enterobacterial repetitive intergenic consensus PCR
HPLC-MS/MS: High performance liquid chromatography - tandem mass spectrometry
Rep-PCR: Repetitive elements sequence-based PCR
