# Supplementary figures and images for: Occurrence, diversity and community structure of culturable atrazine degraders in industrial and agricultural soils exposed to the herbicide in Shandong Province, P.R. China
Source: BMC Microbiol. 2016 Nov 8;16:265. doi: 10.1186/s12866-016-0868-3 (PMC5100194; doi:10.1186/s12866-016-0868-3)

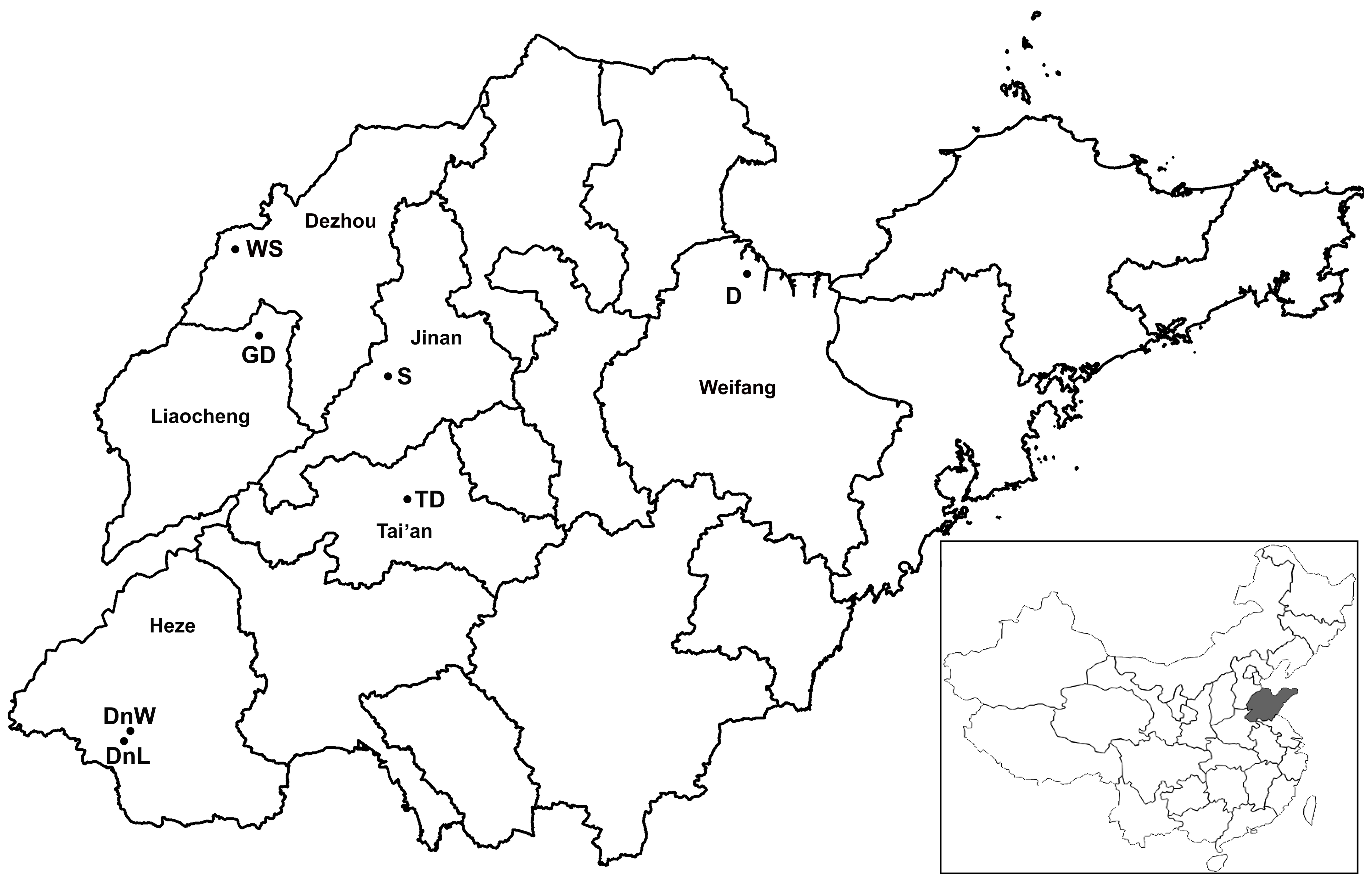

Supplement: Additional file 1: Figure S1. — Map of Shandong Province and location of sampling sites. Location of sampling sites is indicated by the symbol ● with following designations: S - Jinan, Eastern Campus of the Shandong Academy of Sciences; D - Dajiawa Town, Weifang Prefecture; TD - Dongdaguan Village, Dawenkou Township, Tai’an Prefecture; DnW - Wangdian Village, Binhe Township, Dingtao County, Heze Prefecture; DnL - Liulou Village, Binhe Township, Dingtao County, Heze Prefecture; GD - Dawang Village, Guhe Township, Gaotang County, Liaocheng Prefecture; WS - Shadong Village, Guangyun Township, Wucheng County, Dezhou Prefecture. (TIF 1030 kb) [file 12866_2016_868_MOESM1_ESM.tif]

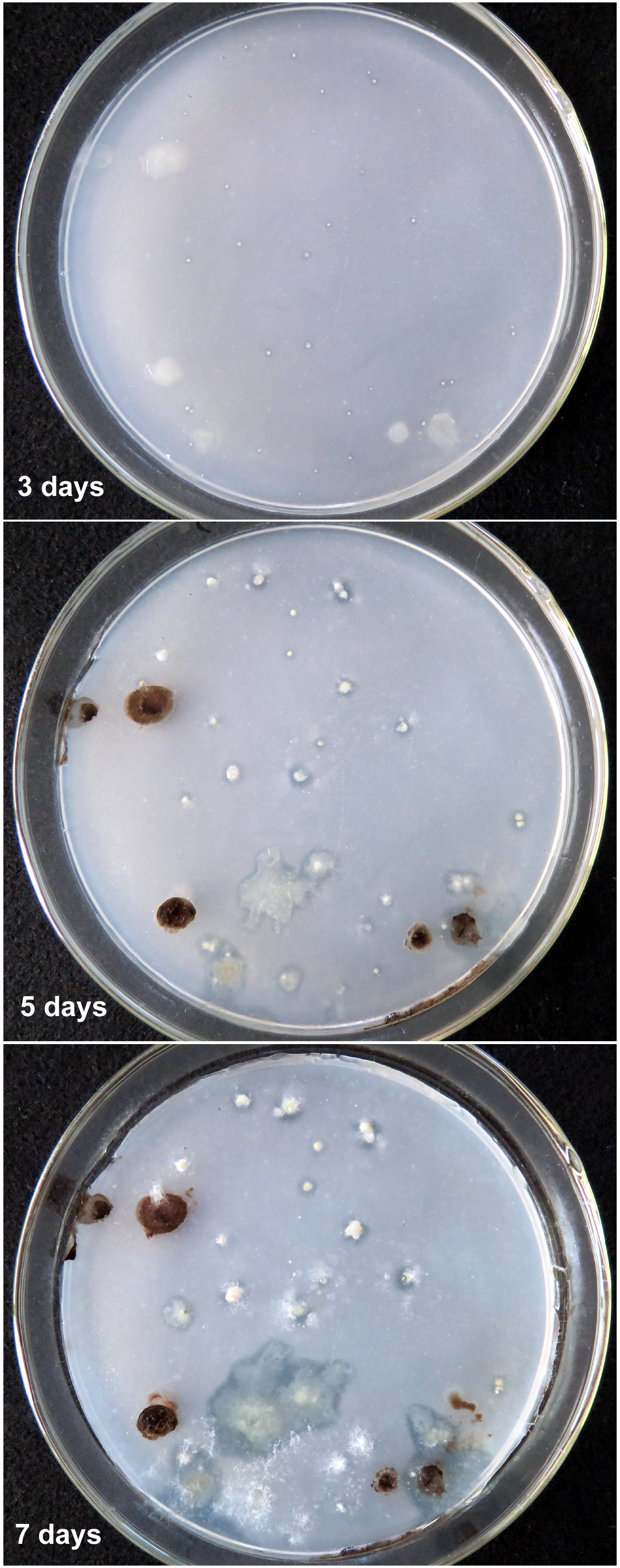

Supplement: Additional file 3: Figure S2. — Direct isolation of atrazine-degrading bacteria on SM agar. 1st dilution of soil suspension (a total of 5 mg soil) was spread on the medium surface. The total population of culturable bacteria was about 109 CFU g−1 soil. The incubation time is indicated near the plates. (TIF 6889 kb) [file 12866_2016_868_MOESM3_ESM.tif]

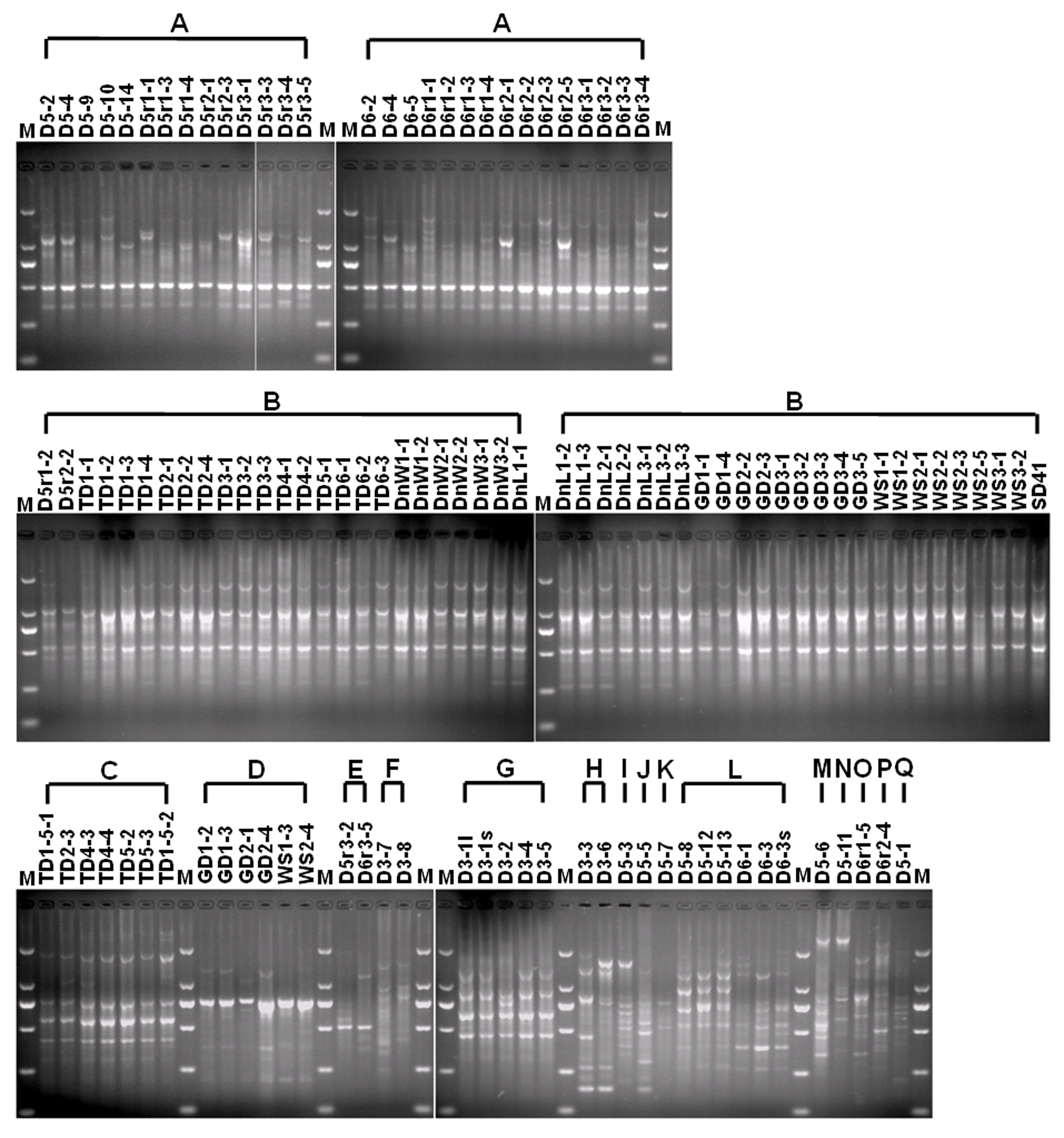

Supplement: Additional file 4: Figure S3. — Discrimination of atrazine-degrading bacteria by ERIC-PCR banding patterns. ERIC types of the bacteria are designated by capital letters under the square brackets. Letters in the name of each strain are designations of the locations as indicated in Table 1. Numbers after letter D represent sampling sites at the location D as listed in Table 1. Letter “r” following D5 and D6 indicates the rhizosphere isolates. The first digit in the names of strains isolated from TD, DnW, DnL, GD and WS sites indicates replicate samples. Site TD(a) was represented by replicate samples 1–3, and site TD(b) by replicate samples 4–6. Lanes M contain DL 2000 DNA Marker (Takara Biotechnology (Dalian) Co., Ltd., China). (TIF 5554 kb) [file 12866_2016_868_MOESM4_ESM.tif]

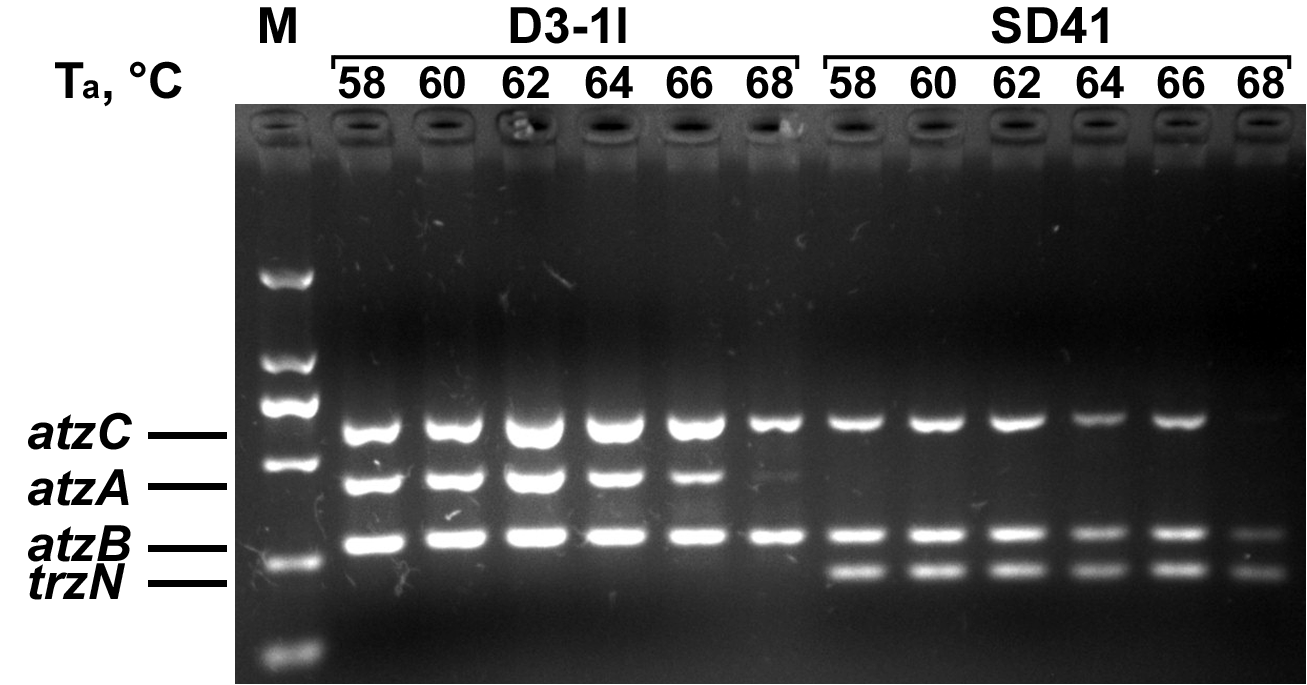

Supplement: Additional file 5: Figure S4. — Amplification products obtained in multiplex gradient PCR targeting trzN, atzA, atzB and atzC with DNAs of the reference strains as templates. Lanes M contain DL 2000 DNA Marker (Takara Biotechnology (Dalian) Co., Ltd., China). (TIF 444 kb) [file 12866_2016_868_MOESM5_ESM.tif]

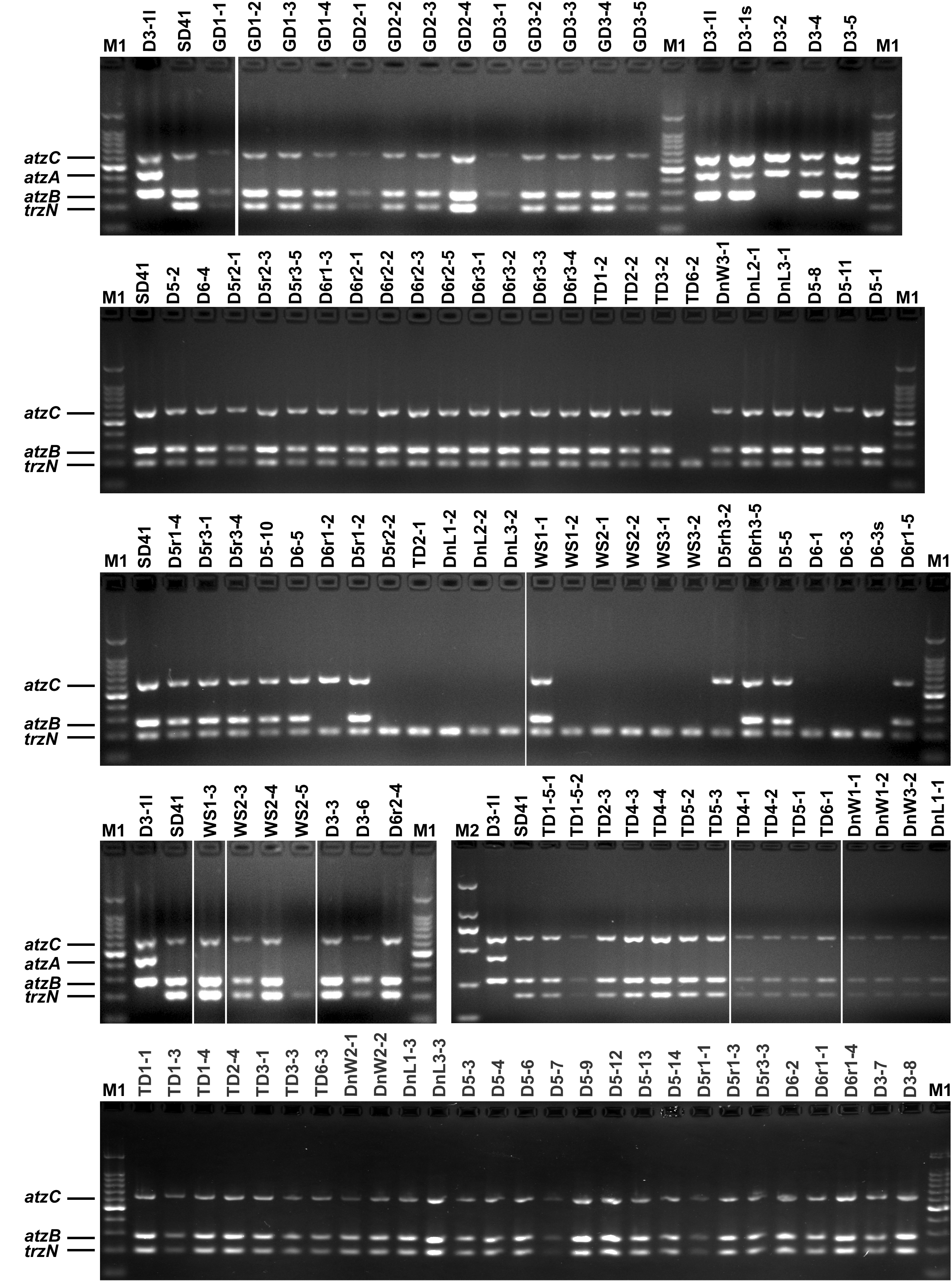

Supplement: Additional file 7: Figure S5. — Detection of the genes for atrazine degradation in isolates by the multiplex PCR assay. Lanes are designated by the isolates’ names. Lanes M1 contain a 100 bp DNA Ladder, lane M2 - DL 2000 DNA Marker (Takara Biotechnology (Dalian) Co., Ltd., China). (TIF 4047 kb) [file 12866_2016_868_MOESM7_ESM.tif]

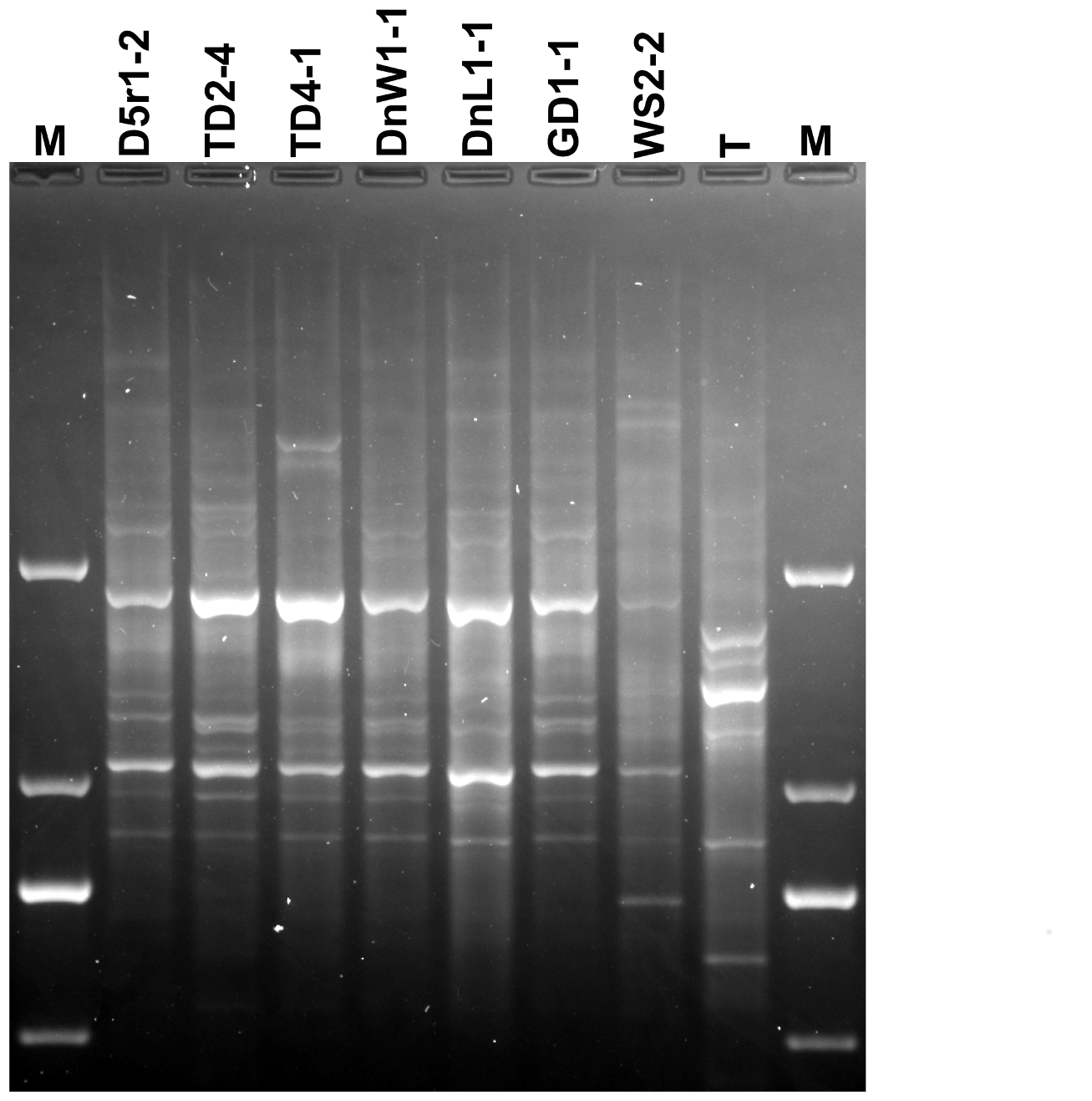

Supplement: Additional file 8: Figure S6. — ERIC-PCR patterns of representative ERIC type B isolates. Lanes are designated by the isolates’ names. Lane T – pattern of the species type strain A. ureafaciens CGMCC 1.1897T. Lane M contains DL 2000 DNA Marker (Takara Biotechnology (Dalian) Co., Ltd., China). (TIF 845 kb) [file 12866_2016_868_MOESM8_ESM.tif]

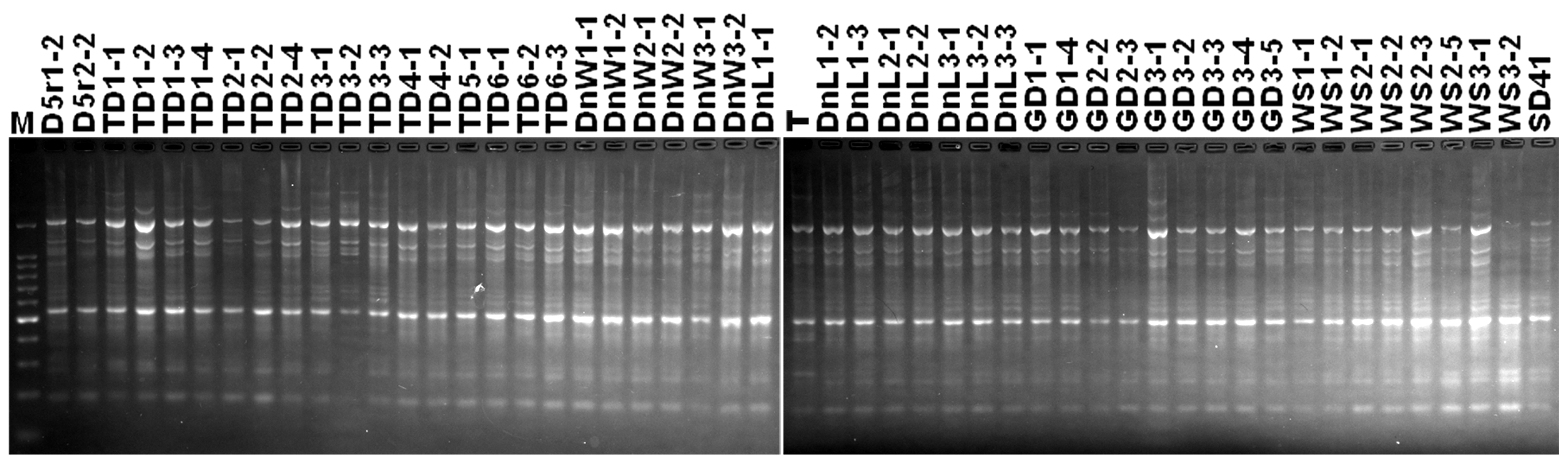

Supplement: Additional file 9: Figure S7. — BOX-PCR typing of ERIC type B isolates. Lanes are designated by the strain names. Lane T – pattern of the species type strain A. ureafaciens CGMCC 1.1897T. Lanes M contain a 100 bp DNA Ladder (Takara Biotechnology (Dalian) Co., Ltd., China). (TIF 1179 kb) [file 12866_2016_868_MOESM9_ESM.tif]

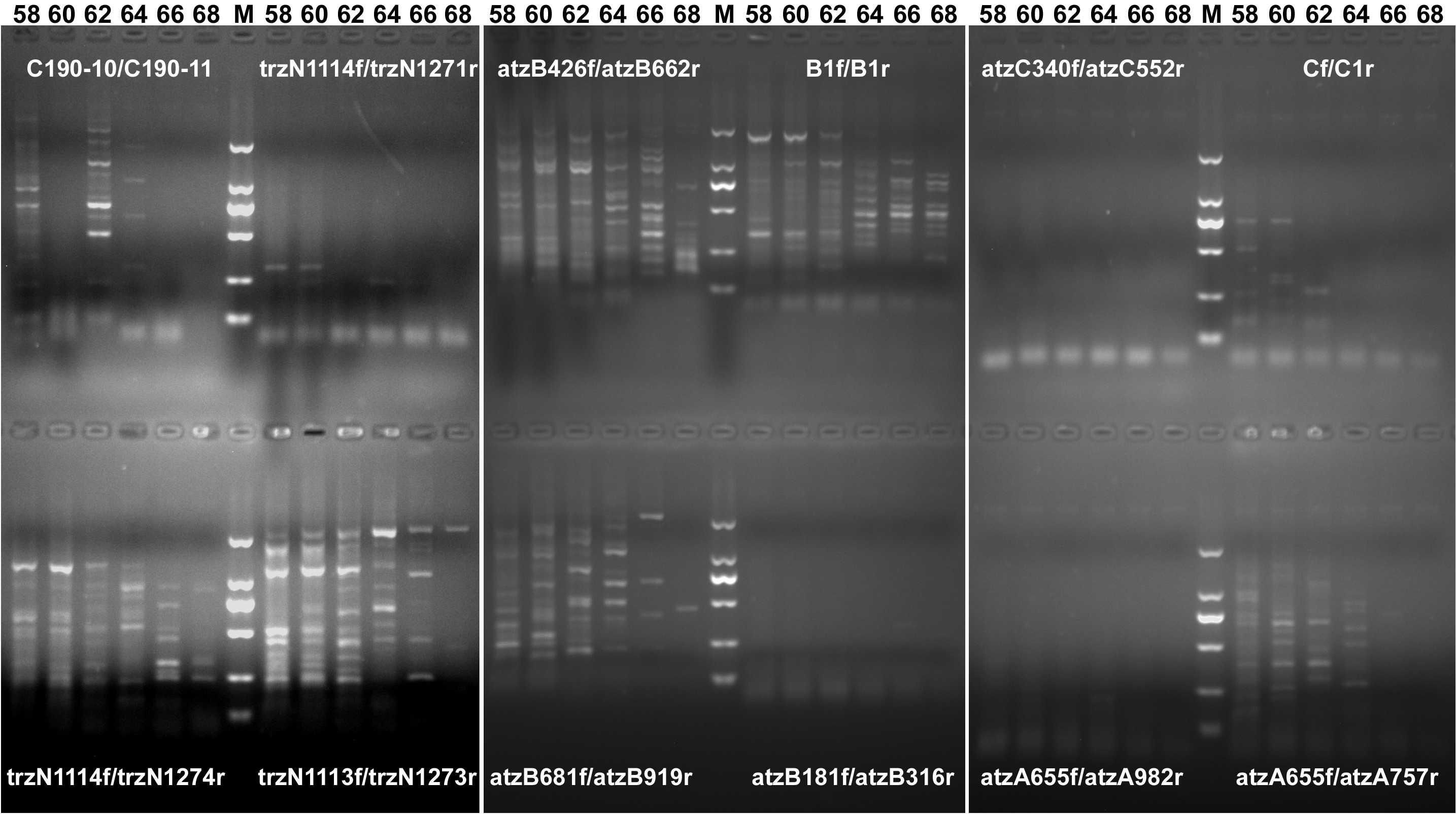

Supplement: Additional file 10: Figure S8. — Amplification of unintended products in PCRs with primers targeting the genes for atrazine degradation. Lanes are designated by values of Ta. Lanes designated by M contain DL 2000 DNA Marker (Takara Biotechnology (Dalian) Co., Ltd., China). The primer pairs are printed on respective gel zones under or below banding patterns. (TIF 2535 kb) [file 12866_2016_868_MOESM10_ESM.tif]

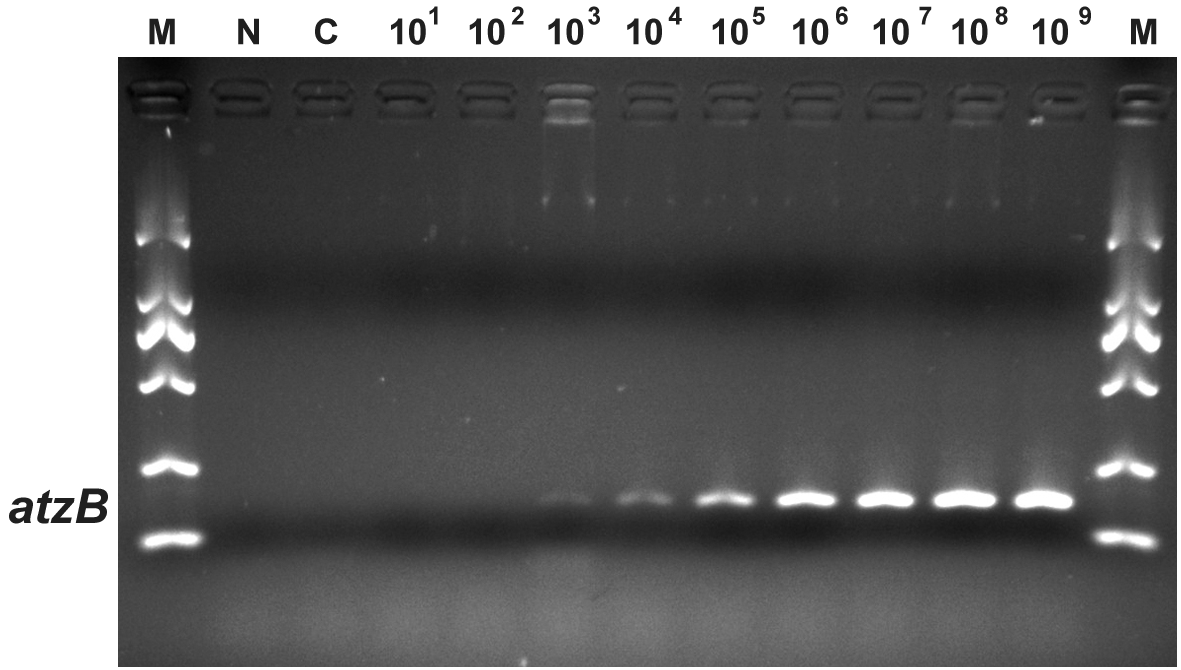

Supplement: Additional file 11: Figure S9. — Detection of the gene atzB in PCRs with template DNAs isolated from S1 soil aliquots with known titers of Pseudomonas sp.D3-1l. (Positive controls for atzB detection results represented in Fig. 4). Lanes are designated by titers of Pseudomonas sp.D3-1l. Lane N and C are, respectively, no template control and a control in which DNA isolated from non-inoculated S1 soil was used as a template. Lanes M contain DL 2000 DNA Marker (Takara Biotechnology (Dalian) Co., Ltd., China). (TIF 385 kb) [file 12866_2016_868_MOESM11_ESM.tif]

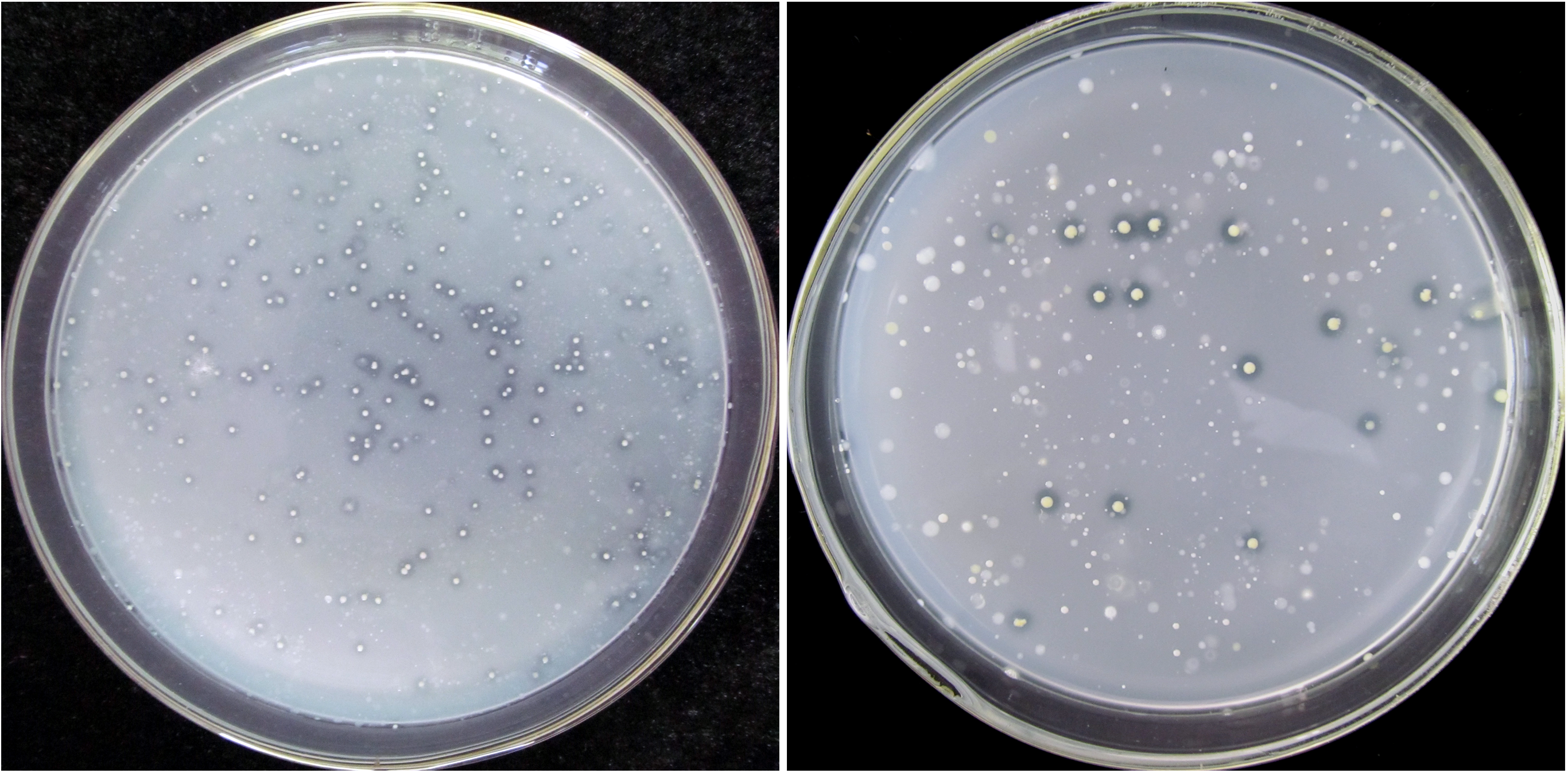

Supplement: Additional file 13: Figure S10. — Recovery of Arthrobacter sp. SD41 from soil by direct plating on SM agar. S1 soil sample was inoculated with about 104 CFU g−1 of Arthrobacter sp.SD41. 1st (left dish) and 2nd (right dish) dilutions of soil suspension were plated on SM agar. Photos of the dishes were taken after 3 (left) and 5 (right) days of incubation at 28 °C. (TIF 5262 kb) [file 12866_2016_868_MOESM13_ESM.tif]
