# Supplementary material for: Occurrence, diversity and community structure of culturable atrazine degraders in industrial and agricultural soils exposed to the herbicide in Shandong Province, P.R. China
Source: BMC Microbiol. 2016 Nov 8;16:265. doi: 10.1186/s12866-016-0868-3 (PMC5100194; doi:10.1186/s12866-016-0868-3)
Supplement: Additional file 2: Table S1. — Soil characteristics. (DOC 50 kb) [file 12866_2016_868_MOESM2_ESM.doc]

**Table S1**. Soil characteristics.

| **Soil** | **Clay (%)** | **Silt (%)** | **Sand (%)** | **pH** | **OC (g/kg)** | **N (mg/kg)** | **P (mg/kg)** | **K (mg/kg)** | **Ca (mg/kg)** | **Mg (mg/kg)** | **Na (mg/kg)** | **Cl (mg/kg)** |
| --- | --- | --- | --- | --- | --- | --- | --- | --- | --- | --- | --- | --- |
| S1 | 13.1 | 85.5 | 1.4 | 7.44 | 16.7 | 67.6±3.4 | 3.4±0.5 | 77±15.4 | 732±73 | 29.1±5.8 | 16±3.2 | 39±0.2 |
| D3 | 8.8 | 73.8 | 17.4 | 7.85 | 5.5 | 437±22 | 10.1±0.5 | 61±12.2 | 661±66 | 10.7±2.1 | 942±94 | 3200±5 |
| D5 | NA | NA | NA | 8.26 | 2.1 | 29.4±1.5 | 8.3±0.5 | 101±20 | 645±65 | 14.7±2.9 | 451±45 | 68±0.3 |
| D6 | 8.7 | 89.0 | 2.3 | 8.11 | 1.3 | 44.2±2.2 | 7.2±0.5 | 89±17.8 | 645±65 | 22.7±4.5 | 446±45 | 680±1.0 |
| TD(a) | 15.5 | 71.1 | 13.4 | 6.10 | 15.1 | 139±7.0 | 48.1±2.4 | 70±14.0 | 676±68 | 18.7±3.7 | 24±4.8 | 87±0.4 |
| TD(b) | 18.3 | 65.4 | 16.3 | 5.93 | 15.2 | 171±8.6 | 52.9±2.7 | 106±21 | 696±70 | 19.0±3.8 | 36±7.2 | 74±0.3 |
| DnW | 14.0 | 84.6 | 1.4 | 7.64 | 10.2 | 79.7±4.0 | 39.0±2.0 | 93±18.6 | 620±62 | 23.5±4.7 | 19±3.8 | 73±0.3 |
| DnL | 14.1 | 84.7 | 1.2 | 7.61 | 8.3 | 89.5±4.5 | 29.0±1.5 | 68±13.6 | 615±62 | 25.3±5.1 | 27±5.4 | 110±0.5 |
| GD | 15.2 | 83.3 | 1.5 | 7.57 | 12.8 | 124±6.2 | 31.2±1.6 | 109±22 | 635±64 | 29.0±5.8 | 75±15.0 | 160±0.7 |
| WS | 17.0 | 81.5 | 1.5 | 7.72 | 9.2 | 160±8.0 | 47.3±2.4 | 92±18.4 | 640±64 | 32.5±6.5 | 73±14.6 | 28±0.1 |

OC – organic carbon, NA – not analyzed.

The standard analysis methods were: NY/T 1377-2007 (**pH**); NY/T 1121.6-2006 (**OC**); HJ 704-2014 (**P**); LY/T 1245-1999 (**Ca** and **Mg**); LY/T 1246-1999 (**Na** and **K**); NY/T 1121.17-2006 (**Cl**). The methods are available at http://www.stdinfo.org.cn. The standard analysis method for **N** was LY/T 1229-1999, available at http://www.csres.com/detail/145787.html.
