# Supplementary material for: Occurrence, diversity and community structure of culturable atrazine degraders in industrial and agricultural soils exposed to the herbicide in Shandong Province, P.R. China
Source: BMC Microbiol. 2016 Nov 8;16:265. doi: 10.1186/s12866-016-0868-3 (PMC5100194; doi:10.1186/s12866-016-0868-3)
Supplement: Additional file 12: — Conditions of HPLC and parameters of MS/MS. (DOC 76 kb) [file 12866_2016_868_MOESM12_ESM.doc]

**Conditions of HPLC and parameters of MS/MS**

**HPLC Conditions**

| Column | Thermo Scientific Syncronis HILIC 250 × 4.6 mm, particle size 5 μm | | |
| --- | --- | --- | --- |
| Solvent A: | 20 mM Ammonium Formate (HPLC Grade, Sinopharm, China) | | |
| Solvent B: | Acetonitrile (HPLC Grade, Sinopharm, China) | | |
| Flow rate: | 800 µL/min | | |
| Injection volume: | 10 µL | | |
| HPLC Gradient: | Time, min | %A | %B |
|  | 0 | 5 | 95 |
|  | 2.0 | 5 | 95 |
|  | 2.1 | 80 | 20 |
|  | 8.0 | 80 | 20 |
|  | 8.1 | 5 | 95 |
|  | 10 | 5 | 95 |

Mass-spectrometer conditions

| Ionization source: | Positive Electrospray (ESI+) |
| --- | --- |
| Sheath gas (N_2_): | 60 arbitrary units |
| Auxiliary gas (N_2_): | 20 arbitrary units |
| ESI voltage: | 3.5 kV |
| Ion transfer tube temperature: | 350°C |
| Collision gas (Ar): | 1.5 mTorr |
| Q1/Q3 peak resolution: | 0.7 Da |
| Scan width: | 0.01 Da |
| Scan dwell time | 0.1 s |

**MS Parameters**

| Compound | Retention time, min | Precursor ion (*m/z*) | Product ion (*m/z*) | Collision energy (eV) | S-lens offset |
| --- | --- | --- | --- | --- | --- |
| Atrazine | 3.92 | 216 | 174 | 18 | 100 |
|  |  | 216 | 104 | 29 | 100 |
|  |  | 216 | 68 | 34 | 100 |


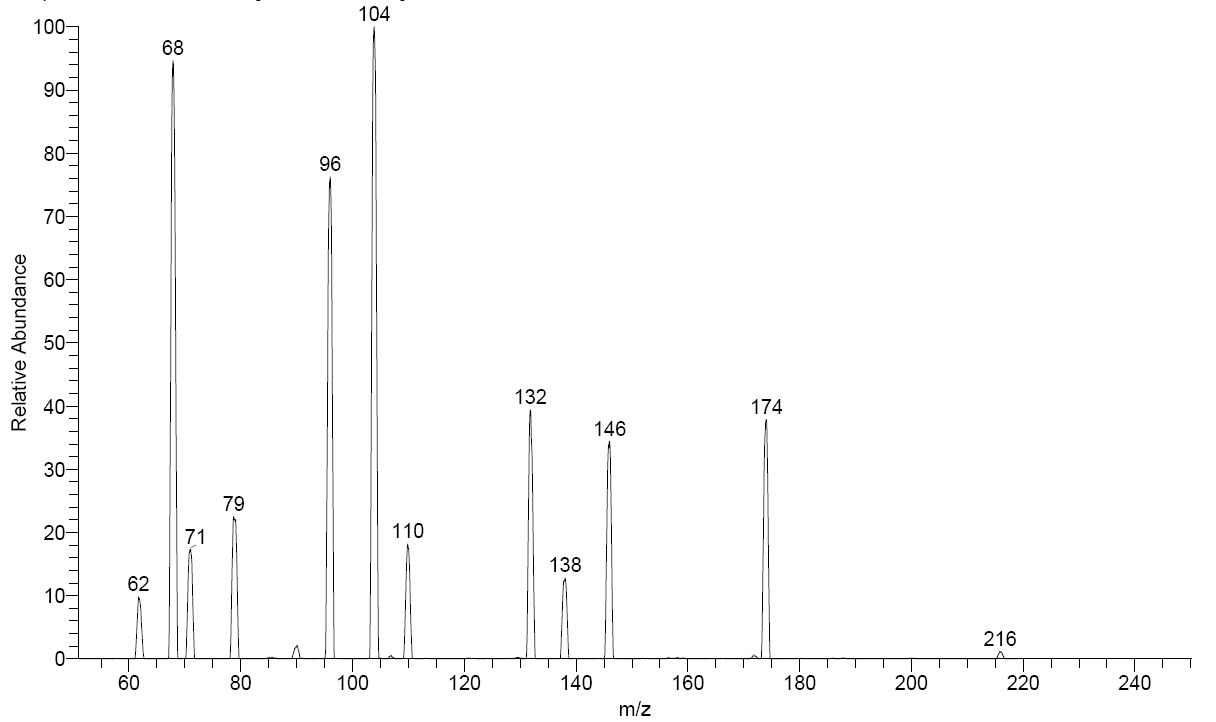


**Fig. Product ion spectrum of atrazine at collision energy 29 eV.**
